# Supplementary material for: Relationship of micro-RNA, mRNA and eIF Expression in Tamoxifen-Adapted MCF-7 Breast Cancer Cells: Impact of miR-1972 on Gene Expression, Proliferation and Migration
Source: Biomolecules. 2022 Jun 29;12(7):916. doi: 10.3390/biom12070916 (PMC9312698; doi:10.3390/biom12070916)
Supplement: Supplementary file 1 [file biomolecules-12-00916-s001.zip › Figure S1.pdf]

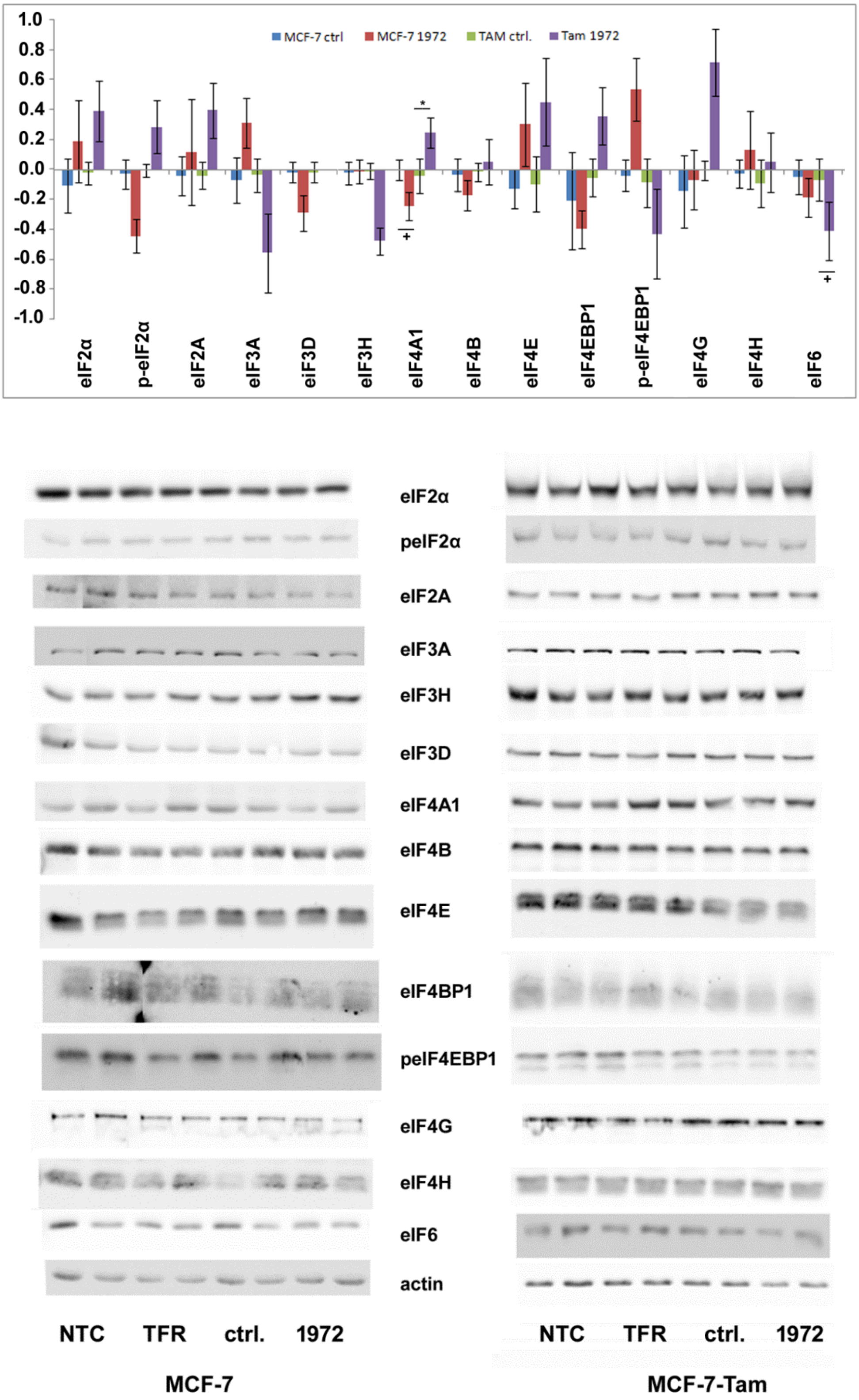

Figure S1: Quantification and statistical analysis (upper panel) as well as representative Western Blot results (lower panel) for the transfection with the miR-1972 mimic (1972) and control (ctrl.) as described in the Materials and Method section. Additionally, untreated cells (NTC) and cells, only incubated with the transfection reagent (TFR), are shown. Two bands each, representing 3 independent experiments with 2 replicas each are shown. Significance was determined by ANOVA and Tamhane T2 post hoc analysis +: $p < 0.1$, *: $p < 0.05$.
