# Supplementary material for: Relationship of micro-RNA, mRNA and eIF Expression in Tamoxifen-Adapted MCF-7 Breast Cancer Cells: Impact of miR-1972 on Gene Expression, Proliferation and Migration
Source: Biomolecules. 2022 Jun 29;12(7):916. doi: 10.3390/biom12070916 (PMC9312698; doi:10.3390/biom12070916)
Supplement: Supplementary file 1 [file biomolecules-12-00916-s001.zip › Table S1 miRNA results.pdf]

microRNA	logFC	AveExpr	t	P.Value	adj.P.Val	B
hsa-miR-181b-5p+hsa-miR-181d-5p	5.91	3.55	44.94	0.00	0.01	5.23
hsa-miR-1972	-3.66	1.46	-33.02	0.00	0.01	4.12
hsa-miR-375	-7.00	2.80	-30.68	0.00	0.01	3.84
hsa-miR-181a-3p	5.85	3.51	21.64	0.00	0.02	2.44
hsa-miR-455-5p	6.38	3.83	17.89	0.00	0.03	1.65
hsa-miR-10a-5p	6.00	3.60	8.81	0.00	0.21	-1.35
hsa-miR-181a-5p	3.09	10.05	8.59	0.00	0.21	-1.46
hsa-miR-135a-5p	-6.21	2.48	-8.33	0.00	0.21	-1.58
hsa-miR-96-5p	-2.12	7.19	-5.43	0.01	0.75	-3.34
hsa-miR-26b-5p	-2.13	8.90	-5.07	0.01	0.76	-3.61
hsa-miR-365a-3p+hsa-miR-365b-3p	-1.71	9.83	-5.08	0.01	0.76	-3.60
hsa-let-7i-5p	-1.22	11.12	-4.08	0.02	0.80	-4.44
hsa-miR-873-3p	3.72	2.92	4.04	0.02	0.80	-4.47
hsa-miR-450a-5p	-2.24	7.72	-3.97	0.02	0.80	-4.54
hsa-miR-98-5p	-1.65	9.86	-4.16	0.02	0.80	-4.36
hsa-miR-193a-5p+hsa-miR-193b-5p	-3.24	1.30	-4.53	0.01	0.80	-4.05
hsa-miR-342-3p	-3.35	10.91	-4.05	0.02	0.80	-4.47
hsa-miR-32-5p	-1.94	10.32	-4.39	0.02	0.80	-4.17
hsa-miR-15b-5p	-1.10	12.48	-4.05	0.02	0.80	-4.47
hsa-miR-1260a	-1.22	7.65	-4.18	0.02	0.80	-4.34
hsa-miR-7-5p	-1.91	9.93	-3.99	0.02	0.80	-4.52
hsa-miR-9-5p	-3.04	6.70	-4.71	0.01	0.80	-3.90
hsa-miR-340-5p	-2.10	7.89	-3.74	0.03	0.91	-4.76
hsa-miR-1257	0.00	0.00	0.00	1.00	1.00	-8.35
hsa-miR-656-3p	0.00	0.00	0.00	1.00	1.00	-8.35
hsa-miR-579-5p	0.00	0.00	0.00	1.00	1.00	-8.35
hsa-miR-574-5p	-0.87	5.19	-1.18	0.31	1.00	-7.60
hsa-miR-4755-5p	0.00	0.00	0.00	1.00	1.00	-8.35
hsa-miR-1269a	0.00	0.00	0.00	1.00	1.00	-8.35

hsa-miR-503-5p	-0.59	7.11	-1.03	0.37	1.00	-7.76
hsa-miR-188-5p	0.24	2.10	0.12	0.91	1.00	-8.35
hsa-miR-138-5p	0.00	0.00	0.00	1.00	1.00	-8.35
hsa-miR-212-3p	0.00	0.00	0.00	1.00	1.00	-8.35
hsa-miR-302b-3p	0.00	0.00	0.00	1.00	1.00	-8.35
hsa-miR-608	0.00	0.00	0.00	1.00	1.00	-8.35
hsa-miR-2053	0.00	0.00	0.00	1.00	1.00	-8.35
hsa-miR-302d-3p	0.00	0.00	0.00	1.00	1.00	-8.35
hsa-miR-28-5p	0.31	8.12	0.44	0.68	1.00	-8.23
hsa-miR-215-5p	0.00	0.00	0.00	1.00	1.00	-8.35
hsa-miR-379-5p	1.01	0.61	0.83	0.46	1.00	-7.95
hsa-miR-30c-5p	-0.33	4.93	-0.35	0.75	1.00	-8.28
hsa-miR-1307-3p	-0.63	1.42	-0.33	0.76	1.00	-8.28
hsa-miR-423-3p	-2.16	3.74	-1.20	0.30	1.00	-7.58
hsa-miR-1258	0.00	0.00	0.00	1.00	1.00	-8.35
hsa-miR-518f-3p	0.00	0.00	0.00	1.00	1.00	-8.35
hsa-miR-593-3p	0.00	0.00	0.00	1.00	1.00	-8.35
hsa-miR-584-5p	0.00	0.00	0.00	1.00	1.00	-8.35
hsa-miR-1234-3p	0.00	0.00	0.00	1.00	1.00	-8.35
hsa-miR-329-5p	0.00	0.00	0.00	1.00	1.00	-8.35
hsa-miR-127-3p	0.00	0.00	0.00	1.00	1.00	-8.35
hsa-miR-1307-5p	0.00	0.00	0.00	1.00	1.00	-8.35
hsa-miR-575	0.00	0.00	0.00	1.00	1.00	-8.35
hsa-miR-125b-5p	0.87	8.98	1.79	0.16	1.00	-6.90
hsa-miR-216b-5p	0.00	0.00	0.00	1.00	1.00	-8.35
hsa-miR-191-5p	-0.13	12.08	-0.18	0.87	1.00	-8.33
hsa-miR-29a-3p	1.19	8.04	2.66	0.07	1.00	-5.87
hsa-miR-1298-5p	0.00	0.00	0.00	1.00	1.00	-8.35
hsa-miR-320a	0.00	0.00	0.00	1.00	1.00	-8.35
hsa-miR-181b-2-3p	0.00	0.00	0.00	1.00	1.00	-8.35

hsa-miR-140-3p	0.00	0.00	0.00	1.00	1.00	-8.35
hsa-miR-149-5p	-0.54	8.27	-0.81	0.47	1.00	-7.97
hsa-miR-371a-5p	0.00	0.00	0.00	1.00	1.00	-8.35
hsa-miR-548i	0.00	0.00	0.00	1.00	1.00	-8.35
hsa-miR-548ah-5p	0.00	0.00	0.00	1.00	1.00	-8.35
hsa-miR-380-3p	0.00	0.00	0.00	1.00	1.00	-8.35
hsa-miR-3934-5p	0.00	0.00	0.00	1.00	1.00	-8.35
hsa-miR-100-5p	0.00	0.00	0.00	1.00	1.00	-8.35
hsa-miR-325	0.00	0.00	0.00	1.00	1.00	-8.35
hsa-miR-3180	0.00	0.00	0.00	1.00	1.00	-8.35
hsa-miR-374a-5p	-1.41	10.77	-2.36	0.09	1.00	-6.21
hsa-miR-651-5p	0.00	0.00	0.00	1.00	1.00	-8.35
hsa-miR-200c-3p	-0.43	12.93	-1.50	0.22	1.00	-7.24
hsa-miR-192-5p	0.00	0.00	0.00	1.00	1.00	-8.35
hsa-miR-770-5p	0.00	0.00	0.00	1.00	1.00	-8.35
hsa-miR-129-2-3p	0.00	0.00	0.00	1.00	1.00	-8.35
hsa-miR-3144-3p	0.00	0.00	0.00	1.00	1.00	-8.35
hsa-miR-6724-5p	0.00	0.00	0.00	1.00	1.00	-8.35
hsa-miR-374a-3p	0.00	0.00	0.00	1.00	1.00	-8.35
hsa-miR-103a-3p	0.00	0.00	0.00	1.00	1.00	-8.35
hsa-miR-549a	0.00	0.00	0.00	1.00	1.00	-8.35
hsa-miR-1236-3p	0.00	0.00	0.00	1.00	1.00	-8.35
hsa-miR-139-3p	0.00	0.00	0.00	1.00	1.00	-8.35
hsa-miR-595	0.00	0.00	0.00	1.00	1.00	-8.35
hsa-miR-1203	0.00	0.00	0.00	1.00	1.00	-8.35
hsa-miR-147a	0.00	0.00	0.00	1.00	1.00	-8.35
hsa-miR-1261	0.00	0.00	0.00	1.00	1.00	-8.35
hsa-miR-519d-3p	0.00	0.00	0.00	1.00	1.00	-8.35
hsa-miR-620	0.00	0.00	0.00	1.00	1.00	-8.35
hsa-miR-561-5p	0.00	0.00	0.00	1.00	1.00	-8.35

hsa-miR-3144-5p	0.00	0.00	0.00	1.00	1.00	-8.35
hsa-miR-644a	0.00	0.00	0.00	1.00	1.00	-8.35
hsa-let-7b-5p	-0.50	11.43	-2.44	0.08	1.00	-6.12
hsa-miR-193a-3p	0.00	0.00	0.00	1.00	1.00	-8.35
hsa-miR-141-3p	-0.73	11.36	-2.35	0.09	1.00	-6.22
hsa-miR-3182	0.00	0.00	0.00	1.00	1.00	-8.35
hsa-miR-339-5p	0.00	0.00	0.00	1.00	1.00	-8.35
hsa-miR-371b-5p	0.00	0.00	0.00	1.00	1.00	-8.35
hsa-miR-92b-3p	0.00	0.00	0.00	1.00	1.00	-8.35
hsa-miR-449c-5p	0.00	0.00	0.00	1.00	1.00	-8.35
hsa-miR-1279	0.00	0.00	0.00	1.00	1.00	-8.35
hsa-miR-155-5p	0.00	0.00	0.00	1.00	1.00	-8.35
hsa-miR-1205	0.00	0.00	0.00	1.00	1.00	-8.35
hsa-miR-941	0.00	0.00	0.00	1.00	1.00	-8.35
hsa-miR-483-3p	0.00	0.00	0.00	1.00	1.00	-8.35
hsa-miR-548ai+hsa-miR-570-5p	0.00	0.00	0.00	1.00	1.00	-8.35
hsa-miR-891b	0.00	0.00	0.00	1.00	1.00	-8.35
hsa-miR-489-3p	-2.74	7.76	-1.90	0.14	1.00	-6.76
hsa-miR-503-3p	0.00	0.00	0.00	1.00	1.00	-8.35
hsa-miR-4451	0.00	0.00	0.00	1.00	1.00	-8.35
hsa-miR-142-3p	-3.00	1.20	-1.44	0.23	1.00	-7.31
hsa-let-7c-5p	0.52	8.64	1.05	0.36	1.00	-7.74
hsa-miR-1193	0.00	0.00	0.00	1.00	1.00	-8.35
hsa-miR-542-5p	0.00	0.00	0.00	1.00	1.00	-8.35
hsa-miR-217	0.00	0.00	0.00	1.00	1.00	-8.35
hsa-miR-125a-3p	0.00	0.00	0.00	1.00	1.00	-8.35
hsa-miR-5196-5p	0.00	0.00	0.00	1.00	1.00	-8.35
hsa-miR-1304-5p	0.00	0.00	0.00	1.00	1.00	-8.35
hsa-miR-520d-3p	0.00	0.00	0.00	1.00	1.00	-8.35
hsa-miR-34b-3p	0.00	0.00	0.00	1.00	1.00	-8.35

hsa-miR-548aa+hsa-miR-548t-3p	0.00	0.00	0.00	1.00	1.00	-8.35
hsa-miR-564	0.00	0.00	0.00	1.00	1.00	-8.35
hsa-miR-488-3p	0.00	0.00	0.00	1.00	1.00	-8.35
hsa-miR-654-5p	0.00	0.00	0.00	1.00	1.00	-8.35
hsa-miR-30b-5p	-0.75	7.46	-1.66	0.18	1.00	-7.05
hsa-miR-592	0.00	0.00	0.00	1.00	1.00	-8.35
hsa-miR-1287-5p	-1.96	0.78	-1.44	0.23	1.00	-7.31
hsa-miR-101-3p	0.00	0.00	0.00	1.00	1.00	-8.35
hsa-miR-509-3p	0.00	0.00	0.00	1.00	1.00	-8.35
hsa-miR-590-5p	-1.72	5.78	-3.47	0.03	1.00	-5.02
hsa-miR-675-5p	0.00	0.00	0.00	1.00	1.00	-8.35
hsa-miR-433-5p	0.00	0.00	0.00	1.00	1.00	-8.35
hsa-miR-571	0.00	0.00	0.00	1.00	1.00	-8.35
hsa-miR-591	0.00	0.00	0.00	1.00	1.00	-8.35
hsa-miR-889-3p	0.00	0.00	0.00	1.00	1.00	-8.35
hsa-miR-587	0.00	0.00	0.00	1.00	1.00	-8.35
hsa-miR-499a-3p	0.00	0.00	0.00	1.00	1.00	-8.35
hsa-miR-1226-3p	0.00	0.00	0.00	1.00	1.00	-8.35
hsa-miR-34c-3p	0.00	0.00	0.00	1.00	1.00	-8.35
hsa-miR-424-5p	-0.92	7.96	-1.85	0.15	1.00	-6.83
hsa-miR-1266-5p	0.00	0.00	0.00	1.00	1.00	-8.35
hsa-miR-520a-5p	0.00	0.00	0.00	1.00	1.00	-8.35
hsa-miR-548ar-5p	0.00	0.00	0.00	1.00	1.00	-8.35
hsa-miR-640	0.00	0.00	0.00	1.00	1.00	-8.35
hsa-miR-877-5p	0.00	0.00	0.00	1.00	1.00	-8.35
hsa-miR-1293	0.00	0.00	0.00	1.00	1.00	-8.35
hsa-miR-515-3p	0.00	0.00	0.00	1.00	1.00	-8.35
hsa-miR-190a-3p	0.00	0.00	0.00	1.00	1.00	-8.35
hsa-miR-302c-3p	0.00	0.00	0.00	1.00	1.00	-8.35
hsa-miR-214-3p	0.00	0.00	0.00	1.00	1.00	-8.35

hsa-miR-516a-3p+hsa-miR-516b-3p	0.00	0.00	0.00	1.00	1.00	-8.35
hsa-miR-555	0.00	0.00	0.00	1.00	1.00	-8.35
hsa-miR-339-3p	0.00	0.00	0.00	1.00	1.00	-8.35
hsa-miR-21-5p	-0.03	15.17	-0.07	0.95	1.00	-8.35
hsa-miR-4531	0.00	0.00	0.00	1.00	1.00	-8.35
hsa-miR-373-3p	0.00	0.00	0.00	1.00	1.00	-8.35
hsa-miR-490-3p	0.00	0.00	0.00	1.00	1.00	-8.35
hsa-miR-1253	0.00	0.00	0.00	1.00	1.00	-8.35
hsa-miR-665	0.00	0.00	0.00	1.00	1.00	-8.35
hsa-miR-4443	1.47	3.13	0.89	0.43	1.00	-7.89
hsa-miR-628-3p	0.00	0.00	0.00	1.00	1.00	-8.35
hsa-miR-616-3p	0.00	0.00	0.00	1.00	1.00	-8.35
hsa-miR-3928-3p	0.00	0.00	0.00	1.00	1.00	-8.35
hsa-miR-196a-3p	0.00	0.00	0.00	1.00	1.00	-8.35
hsa-miR-548al	0.00	0.00	0.00	1.00	1.00	-8.35
hsa-miR-1287-3p	0.00	0.00	0.00	1.00	1.00	-8.35
hsa-miR-1250-5p	0.00	0.00	0.00	1.00	1.00	-8.35
hsa-let-7a-5p	-0.81	13.83	-2.36	0.09	1.00	-6.21
hsa-miR-3158-3p	0.00	0.00	0.00	1.00	1.00	-8.35
hsa-miR-1246	0.00	0.00	0.00	1.00	1.00	-8.35
hsa-miR-190b	0.00	0.00	0.00	1.00	1.00	-8.35
hsa-miR-450b-3p	0.00	0.00	0.00	1.00	1.00	-8.35
hsa-miR-1827	0.00	0.00	0.00	1.00	1.00	-8.35
hsa-miR-423-5p	-0.46	4.76	-0.62	0.57	1.00	-8.12
hsa-miR-499b-3p	0.00	0.00	0.00	1.00	1.00	-8.35
hsa-miR-603	0.00	0.00	0.00	1.00	1.00	-8.35
hsa-miR-885-3p	0.00	0.00	0.00	1.00	1.00	-8.35
hsa-miR-381-3p	0.00	0.00	0.00	1.00	1.00	-8.35
hsa-miR-4536-5p	0.00	0.00	0.00	1.00	1.00	-8.35
hsa-miR-671-5p	0.00	0.00	0.00	1.00	1.00	-8.35

hsa-miR-520c-3p	0.00	0.00	0.00	1.00	1.00	-8.35
hsa-miR-520g-3p	0.00	0.00	0.00	1.00	1.00	-8.35
hsa-miR-2682-5p	0.00	0.00	0.00	1.00	1.00	-8.35
hsa-miR-509-3-5p	0.00	0.00	0.00	1.00	1.00	-8.35
hsa-miR-4787-5p	0.00	0.00	0.00	1.00	1.00	-8.35
hsa-miR-1289	0.00	0.00	0.00	1.00	1.00	-8.35
hsa-miR-1268a	0.00	0.00	0.00	1.00	1.00	-8.35
hsa-miR-604	0.00	0.00	0.00	1.00	1.00	-8.35
hsa-miR-302e	0.00	0.00	0.00	1.00	1.00	-8.35
hsa-miR-19a-3p	-1.12	6.16	-1.05	0.36	1.00	-7.74
hsa-miR-122-5p	0.00	0.00	0.00	1.00	1.00	-8.35
hsa-miR-499b-5p	0.00	0.00	0.00	1.00	1.00	-8.35
hsa-miR-1273c	0.00	0.00	0.00	1.00	1.00	-8.35
hsa-miR-1206	-2.83	1.13	-1.44	0.23	1.00	-7.31
hsa-miR-513a-5p	0.00	0.00	0.00	1.00	1.00	-8.35
hsa-miR-589-5p	0.00	0.00	0.00	1.00	1.00	-8.35
hsa-miR-382-5p	0.00	0.00	0.00	1.00	1.00	-8.35
hsa-miR-3180-3p	0.00	0.00	0.00	1.00	1.00	-8.35
hsa-miR-758-3p+hsa-miR-411-3p	0.00	0.00	0.00	1.00	1.00	-8.35
hsa-miR-376a-2-5p	0.00	0.00	0.00	1.00	1.00	-8.35
hsa-miR-577	0.00	0.00	0.00	1.00	1.00	-8.35
hsa-miR-135b-5p	-2.83	1.13	-1.44	0.23	1.00	-7.31
hsa-miR-140-5p	-2.04	5.01	-2.37	0.09	1.00	-6.21
hsa-miR-5196-3p+hsa-miR-6732-3p	-1.73	0.69	-1.44	0.23	1.00	-7.31
hsa-miR-6721-5p	2.26	1.36	1.30	0.27	1.00	-7.47
hsa-miR-1202	0.00	0.00	0.00	1.00	1.00	-8.35
hsa-miR-148a-3p	-1.61	8.89	-2.87	0.05	1.00	-5.64
hsa-miR-190a-5p	-1.26	0.50	-1.44	0.23	1.00	-7.31
hsa-miR-139-5p	0.00	0.00	0.00	1.00	1.00	-8.35
hsa-miR-216a-5p	0.00	0.00	0.00	1.00	1.00	-8.35

hsa-miR-302f	0.00	0.00	0.00	1.00	1.00	-8.35
hsa-miR-548b-3p	0.00	0.00	0.00	1.00	1.00	-8.35
hsa-miR-541-3p	0.00	0.00	0.00	1.00	1.00	-8.35
hsa-miR-372-3p	0.00	0.00	0.00	1.00	1.00	-8.35
hsa-miR-324-5p	-1.22	7.29	-3.12	0.04	1.00	-5.37
hsa-miR-3202	0.00	0.00	0.00	1.00	1.00	-8.35
hsa-miR-409-3p	0.00	0.00	0.00	1.00	1.00	-8.35
hsa-miR-492	0.00	0.00	0.00	1.00	1.00	-8.35
hsa-miR-500a-5p+hsa-miR-501-5p	-0.06	2.39	-0.03	0.98	1.00	-8.35
hsa-miR-1469	0.00	0.00	0.00	1.00	1.00	-8.35
hsa-miR-328-3p	0.00	0.00	0.00	1.00	1.00	-8.35
hsa-miR-940	0.00	0.00	0.00	1.00	1.00	-8.35
hsa-miR-31-5p	0.00	0.00	0.00	1.00	1.00	-8.35
hsa-miR-518b	0.00	0.00	0.00	1.00	1.00	-8.35
hsa-miR-615-5p	0.00	0.00	0.00	1.00	1.00	-8.35
hsa-miR-1276	0.00	0.00	0.00	1.00	1.00	-8.35
hsa-miR-548e-3p	0.00	0.00	0.00	1.00	1.00	-8.35
hsa-miR-1908-3p	0.00	0.00	0.00	1.00	1.00	-8.35
hsa-miR-3150b-3p	0.00	0.00	0.00	1.00	1.00	-8.35
hsa-miR-504-5p	1.33	0.80	0.83	0.46	1.00	-7.95
hsa-miR-619-3p	0.00	0.00	0.00	1.00	1.00	-8.35
hsa-miR-363-5p	0.00	0.00	0.00	1.00	1.00	-8.35
hsa-miR-4435	0.00	0.00	0.00	1.00	1.00	-8.35
hsa-miR-890	0.00	0.00	0.00	1.00	1.00	-8.35
hsa-miR-520f-3p	0.00	0.00	0.00	1.00	1.00	-8.35
hsa-miR-151a-3p	-0.12	8.11	-0.21	0.84	1.00	-8.33
hsa-miR-194-5p	0.32	6.62	0.58	0.60	1.00	-8.15
hsa-miR-218-5p	-3.12	4.20	-1.62	0.19	1.00	-7.10
hsa-miR-15a-5p	-0.57	11.85	-1.49	0.22	1.00	-7.25
hsa-miR-539-5p	0.00	0.00	0.00	1.00	1.00	-8.35

hsa-miR-493-3p	0.00	0.00	0.00	1.00	1.00	-8.35
hsa-miR-4454+hsa-miR-7975	-0.22	12.79	-0.51	0.64	1.00	-8.19
hsa-miR-3161	0.00	0.00	0.00	1.00	1.00	-8.35
hsa-miR-4521	0.00	0.00	0.00	1.00	1.00	-8.35
hsa-miR-422a	0.00	0.00	0.00	1.00	1.00	-8.35
hsa-miR-374c-5p	0.00	0.00	0.00	1.00	1.00	-8.35
hsa-miR-551a	0.00	0.00	0.00	1.00	1.00	-8.35
hsa-miR-874-5p	0.00	0.00	0.00	1.00	1.00	-8.35
hsa-miR-1180-3p	-0.18	8.47	-1.38	0.25	1.00	-7.38
hsa-miR-579-3p	0.00	0.00	0.00	1.00	1.00	-8.35
hsa-miR-128-1-5p	0.00	0.00	0.00	1.00	1.00	-8.35
hsa-miR-610	0.00	0.00	0.00	1.00	1.00	-8.35
hsa-miR-3136-5p	0.00	0.00	0.00	1.00	1.00	-8.35
hsa-miR-671-3p	0.00	0.00	0.00	1.00	1.00	-8.35
hsa-miR-219a-1-3p	0.00	0.00	0.00	1.00	1.00	-8.35
hsa-miR-1291	0.00	0.00	0.00	1.00	1.00	-8.35
hsa-miR-342-5p	-2.92	1.17	-1.44	0.23	1.00	-7.31
hsa-miR-320b	0.00	0.00	0.00	1.00	1.00	-8.35
hsa-miR-195-5p	-2.56	2.16	-1.38	0.25	1.00	-7.38
hsa-miR-202-3p	0.00	0.00	0.00	1.00	1.00	-8.35
hsa-miR-152-3p	-1.91	0.76	-1.44	0.23	1.00	-7.31
hsa-miR-519b-5p+hsa-miR-519c-5p+ hsa-miR-523-5p+hsa-miR-518e-5p+ hsa-miR-522-5p+hsa-miR-519a-5p	0.00	0.00	0.00	1.00	1.00	-8.35
hsa-miR-617	0.00	0.00	0.00	1.00	1.00	-8.35
hsa-miR-374b-5p	-0.96	8.42	-1.87	0.15	1.00	-6.80
hsa-miR-92a-3p	-1.14	3.56	-0.65	0.55	1.00	-8.10
hsa-miR-2117	0.00	0.00	0.00	1.00	1.00	-8.35
hsa-miR-567	0.00	0.00	0.00	1.00	1.00	-8.35
hsa-miR-24-3p	-0.48	8.58	-0.91	0.42	1.00	-7.88

hsa-miR-524-3p	0.00	0.00	0.00	1.00	1.00	-8.35
hsa-miR-515-5p	0.00	0.00	0.00	1.00	1.00	-8.35
hsa-miR-4458	0.00	0.00	0.00	1.00	1.00	-8.35
hsa-miR-3164	0.00	0.00	0.00	1.00	1.00	-8.35
hsa-miR-519e-3p	0.00	0.00	0.00	1.00	1.00	-8.35
hsa-miR-98-3p	0.00	0.00	0.00	1.00	1.00	-8.35
hsa-miR-767-3p	0.00	0.00	0.00	1.00	1.00	-8.35
hsa-miR-606	0.00	0.00	0.00	1.00	1.00	-8.35
hsa-miR-365b-5p	0.00	0.00	0.00	1.00	1.00	-8.35
hsa-miR-3690	0.00	0.00	0.00	1.00	1.00	-8.35
hsa-miR-505-3p	-1.39	3.86	-0.60	0.59	1.00	-8.13
hsa-miR-1264	0.00	0.00	0.00	1.00	1.00	-8.35
hsa-miR-519b-3p	0.00	0.00	0.00	1.00	1.00	-8.35
hsa-miR-596	0.00	0.00	0.00	1.00	1.00	-8.35
hsa-miR-105-5p	0.00	0.00	0.00	1.00	1.00	-8.35
hsa-miR-576-5p	0.00	0.00	0.00	1.00	1.00	-8.35
hsa-miR-892a	0.00	0.00	0.00	1.00	1.00	-8.35
hsa-miR-150-5p	0.00	0.00	0.00	1.00	1.00	-8.35
hsa-miR-520d-5p+hsa-miR-527+ hsa-miR-518a-5p	0.00	0.00	0.00	1.00	1.00	-8.35
hsa-miR-19b-3p	-0.29	9.08	-0.48	0.66	1.00	-8.21
hsa-miR-370-5p	0.00	0.00	0.00	1.00	1.00	-8.35
hsa-miR-3918	0.00	0.00	0.00	1.00	1.00	-8.35
hsa-miR-548n	0.00	0.00	0.00	1.00	1.00	-8.35
hsa-miR-1244	0.00	0.00	0.00	1.00	1.00	-8.35
hsa-miR-320c	0.00	0.00	0.00	1.00	1.00	-8.35
hsa-miR-345-5p	-2.01	0.80	-1.44	0.23	1.00	-7.31
hsa-miR-548d-5p	-1.26	0.50	-1.44	0.23	1.00	-7.31
hsa-miR-29b-3p	-0.20	10.31	-0.49	0.65	1.00	-8.20
hsa-miR-93-5p	-0.44	12.58	-1.56	0.20	1.00	-7.17

hsa-miR-383-5p	0.00	0.00	0.00	1.00	1.00	-8.35
hsa-miR-516b-5p	0.00	0.00	0.00	1.00	1.00	-8.35
hsa-miR-613	0.00	0.00	0.00	1.00	1.00	-8.35
hsa-miR-1248	0.00	0.00	0.00	1.00	1.00	-8.35
hsa-miR-935	0.00	0.00	0.00	1.00	1.00	-8.35
hsa-miR-548j-3p	0.00	0.00	0.00	1.00	1.00	-8.35
hsa-miR-3074-3p	0.00	0.00	0.00	1.00	1.00	-8.35
hsa-miR-450a-1-3p	0.00	0.00	0.00	1.00	1.00	-8.35
hsa-miR-568	0.00	0.00	0.00	1.00	1.00	-8.35
hsa-miR-509-5p	0.00	0.00	0.00	1.00	1.00	-8.35
hsa-miR-525-3p	0.00	0.00	0.00	1.00	1.00	-8.35
hsa-miR-143-3p	0.00	0.00	0.00	1.00	1.00	-8.35
hsa-miR-151b	0.00	0.00	0.00	1.00	1.00	-8.35
hsa-miR-326	0.00	0.00	0.00	1.00	1.00	-8.35
hsa-miR-20a-5p+hsa-miR-20b-5p	-0.38	9.36	-0.74	0.51	1.00	-8.03
hsa-miR-208b-5p	0.00	0.00	0.00	1.00	1.00	-8.35
hsa-miR-142-5p	0.00	0.00	0.00	1.00	1.00	-8.35
hsa-miR-367-3p	0.00	0.00	0.00	1.00	1.00	-8.35
hsa-miR-556-3p	0.00	0.00	0.00	1.00	1.00	-8.35
hsa-miR-320d	0.00	0.00	0.00	1.00	1.00	-8.35
hsa-miR-384	0.00	0.00	0.00	1.00	1.00	-8.35
hsa-miR-548a-3p	0.00	0.00	0.00	1.00	1.00	-8.35
hsa-miR-523-3p	0.00	0.00	0.00	1.00	1.00	-8.35
hsa-miR-491-3p	0.00	0.00	0.00	1.00	1.00	-8.35
hsa-miR-3195	0.00	0.00	0.00	1.00	1.00	-8.35
hsa-miR-323b-3p	0.00	0.00	0.00	1.00	1.00	-8.35
hsa-miR-1285-3p	0.00	0.00	0.00	1.00	1.00	-8.35
hsa-miR-221-3p	2.36	1.41	1.14	0.33	1.00	-7.65
hsa-miR-376a-3p	0.00	0.00	0.00	1.00	1.00	-8.35
hsa-miR-526a+hsa-miR-518c-5p+	0.00	0.00	0.00	1.00	1.00	-8.35

hsa-miR-518d-5p						
hsa-let-7d-5p	-0.52	12.32	-1.38	0.25	1.00	-7.37
hsa-miR-196a-5p	0.54	6.43	0.57	0.60	1.00	-8.15
hsa-miR-1299	0.00	0.00	0.00	1.00	1.00	-8.35
hsa-miR-506-3p	0.00	0.00	0.00	1.00	1.00	-8.35
hsa-miR-491-5p	0.00	0.00	0.00	1.00	1.00	-8.35
hsa-miR-513b-5p	0.00	0.00	0.00	1.00	1.00	-8.35
hsa-miR-514a-5p	0.00	0.00	0.00	1.00	1.00	-8.35
hsa-miR-1183	0.00	0.00	0.00	1.00	1.00	-8.35
hsa-miR-1910-5p	0.00	0.00	0.00	1.00	1.00	-8.35
hsa-miR-451a	0.00	0.00	0.00	1.00	1.00	-8.35
hsa-miR-3180-5p	0.00	0.00	0.00	1.00	1.00	-8.35
hsa-miR-6503-3p	0.00	0.00	0.00	1.00	1.00	-8.35
hsa-miR-193b-3p	-3.79	3.62	-2.00	0.13	1.00	-6.64
hsa-miR-144-3p	0.00	0.00	0.00	1.00	1.00	-8.35
hsa-miR-4421	0.00	0.00	0.00	1.00	1.00	-8.35
hsa-miR-873-5p	0.00	0.00	0.00	1.00	1.00	-8.35
hsa-miR-942-3p	0.00	0.00	0.00	1.00	1.00	-8.35
hsa-miR-532-5p	-2.34	0.94	-1.44	0.23	1.00	-7.31
hsa-miR-320e	2.96	4.57	1.50	0.22	1.00	-7.24
hsa-miR-29c-3p	-0.64	8.31	-1.14	0.33	1.00	-7.64
hsa-miR-219a-2-3p	0.00	0.00	0.00	1.00	1.00	-8.35
hsa-miR-875-3p	0.00	0.00	0.00	1.00	1.00	-8.35
hsa-miR-145-5p	0.00	0.00	0.00	1.00	1.00	-8.35
hsa-miR-1204	0.00	0.00	0.00	1.00	1.00	-8.35
hsa-miR-429	-2.09	6.00	-2.83	0.06	1.00	-5.69
hsa-miR-570-3p	0.00	0.00	0.00	1.00	1.00	-8.35
hsa-miR-578	0.00	0.00	0.00	1.00	1.00	-8.35
hsa-miR-490-5p	0.00	0.00	0.00	1.00	1.00	-8.35
hsa-miR-1245b-3p	0.00	0.00	0.00	1.00	1.00	-8.35

hsa-miR-34c-5p	0.00	0.00	0.00	1.00	1.00	-8.35
hsa-miR-381-5p	0.00	0.00	0.00	1.00	1.00	-8.35
hsa-miR-550a-5p	0.00	0.00	0.00	1.00	1.00	-8.35
hsa-miR-1233-3p	0.00	0.00	0.00	1.00	1.00	-8.35
hsa-miR-518c-3p	0.00	0.00	0.00	1.00	1.00	-8.35
hsa-miR-4524a-5p	0.00	0.00	0.00	1.00	1.00	-8.35
hsa-miR-580-3p	0.00	0.00	0.00	1.00	1.00	-8.35
hsa-miR-944	0.00	0.00	0.00	1.00	1.00	-8.35
hsa-miR-152-5p	0.00	0.00	0.00	1.00	1.00	-8.35
hsa-miR-219a-5p	0.00	0.00	0.00	1.00	1.00	-8.35
hsa-miR-153-3p	0.00	0.00	0.00	1.00	1.00	-8.35
hsa-miR-124-3p	0.00	0.00	0.00	1.00	1.00	-8.35
hsa-miR-5010-5p	0.00	0.00	0.00	1.00	1.00	-8.35
hsa-miR-545-3p	-1.57	0.63	-1.44	0.23	1.00	-7.31
hsa-miR-654-3p	0.00	0.00	0.00	1.00	1.00	-8.35
hsa-miR-1224-5p	0.00	0.00	0.00	1.00	1.00	-8.35
hsa-miR-3192-5p	0.00	0.00	0.00	1.00	1.00	-8.35
hsa-miR-425-5p	-0.89	7.51	-2.47	0.08	1.00	-6.10
hsa-miR-561-3p	0.00	0.00	0.00	1.00	1.00	-8.35
hsa-miR-553	0.00	0.00	0.00	1.00	1.00	-8.35
hsa-miR-516a-5p	0.00	0.00	0.00	1.00	1.00	-8.35
hsa-miR-1271-3p	0.00	0.00	0.00	1.00	1.00	-8.35
hsa-miR-601	0.00	0.00	0.00	1.00	1.00	-8.35
hsa-miR-1288-3p	0.00	0.00	0.00	1.00	1.00	-8.35
hsa-miR-133b	0.00	0.00	0.00	1.00	1.00	-8.35
hsa-miR-504-3p	0.00	0.00	0.00	1.00	1.00	-8.35
hsa-miR-4787-3p	0.00	0.00	0.00	1.00	1.00	-8.35
hsa-miR-708-5p	0.00	0.00	0.00	1.00	1.00	-8.35
hsa-miR-329-3p	0.00	0.00	0.00	1.00	1.00	-8.35
hsa-miR-548o-3p+hsa-miR-548ah-	0.00	0.00	0.00	1.00	1.00	-8.35

3p+hsa-miR-548av-3p						
hsa-miR-431-5p	0.00	0.00	0.00	1.00	1.00	-8.35
hsa-miR-1-3p	0.00	0.00	0.00	1.00	1.00	-8.35
hsa-miR-887-3p	0.00	0.00	0.00	1.00	1.00	-8.35
hsa-miR-127-5p	0.00	0.00	0.00	1.00	1.00	-8.35
hsa-miR-3179	0.00	0.00	0.00	1.00	1.00	-8.35
hsa-miR-499a-5p	0.00	0.00	0.00	1.00	1.00	-8.35
hsa-miR-1908-5p	0.00	0.00	0.00	1.00	1.00	-8.35
hsa-miR-518d-3p	0.00	0.00	0.00	1.00	1.00	-8.35
hsa-miR-612	-1.76	0.86	-1.31	0.27	1.00	-7.46
hsa-miR-450b-5p	-1.96	0.78	-1.44	0.23	1.00	-7.31
hsa-miR-3614-3p	0.00	0.00	0.00	1.00	1.00	-8.35
hsa-miR-548l	0.00	0.00	0.00	1.00	1.00	-8.35
hsa-miR-1306-5p	0.00	0.00	0.00	1.00	1.00	-8.35
hsa-miR-3196	0.00	0.00	0.00	1.00	1.00	-8.35
hsa-miR-130b-3p	-0.31	0.12	-1.44	0.23	1.00	-7.31
hsa-miR-1910-3p	0.00	0.00	0.00	1.00	1.00	-8.35
hsa-miR-1247-5p	0.00	0.00	0.00	1.00	1.00	-8.35
hsa-miR-151a-5p	0.67	5.25	0.42	0.70	1.00	-8.25
hsa-miR-3140-3p	0.00	0.00	0.00	1.00	1.00	-8.35
hsa-miR-211-5p	0.00	0.00	0.00	1.00	1.00	-8.35
hsa-miR-23b-3p	0.86	10.58	1.32	0.27	1.00	-7.44
hsa-miR-526b-5p	0.00	0.00	0.00	1.00	1.00	-8.35
hsa-miR-337-5p	0.00	0.00	0.00	1.00	1.00	-8.35
hsa-miR-369-3p	0.00	0.00	0.00	1.00	1.00	-8.35
hsa-miR-28-3p	0.61	2.42	0.27	0.80	1.00	-8.31
hsa-miR-585-3p	0.00	0.00	0.00	1.00	1.00	-8.35
hsa-miR-185-5p	-0.62	5.76	-1.11	0.34	1.00	-7.67
hsa-miR-497-5p	-2.62	6.15	-2.47	0.08	1.00	-6.09
hsa-miR-1323	0.00	0.00	0.00	1.00	1.00	-8.35

hsa-miR-1262	0.00	0.00	0.00	1.00	1.00	-8.35
hsa-miR-512-5p	0.00	0.00	0.00	1.00	1.00	-8.35
hsa-miR-4536-3p	0.00	0.00	0.00	1.00	1.00	-8.35
hsa-miR-33b-5p	0.00	0.00	0.00	1.00	1.00	-8.35
hsa-miR-548a-5p	0.00	0.00	0.00	1.00	1.00	-8.35
hsa-miR-582-5p	-2.28	0.91	-1.44	0.23	1.00	-7.31
hsa-miR-508-5p	0.00	0.00	0.00	1.00	1.00	-8.35
hsa-miR-626	0.00	0.00	0.00	1.00	1.00	-8.35
hsa-miR-30e-3p	-2.40	0.96	-1.44	0.23	1.00	-7.31
hsa-miR-132-3p	0.72	6.04	0.88	0.44	1.00	-7.91
hsa-miR-203a-5p	0.00	0.00	0.00	1.00	1.00	-8.35
hsa-miR-6503-5p	0.00	0.00	0.00	1.00	1.00	-8.35
hsa-miR-1278	0.00	0.00	0.00	1.00	1.00	-8.35
hsa-miR-922	0.00	0.00	0.00	1.00	1.00	-8.35
hsa-miR-338-5p	0.00	0.00	0.00	1.00	1.00	-8.35
hsa-miR-23c	0.00	0.00	0.00	1.00	1.00	-8.35
hsa-miR-4284	0.00	0.00	0.00	1.00	1.00	-8.35
hsa-miR-769-3p	0.00	0.00	0.00	1.00	1.00	-8.35
hsa-miR-369-5p	0.00	0.00	0.00	1.00	1.00	-8.35
hsa-miR-18a-5p	-1.01	6.07	-1.08	0.35	1.00	-7.71
hsa-miR-126-3p	0.37	9.11	0.69	0.54	1.00	-8.07
hsa-miR-1185-2-3p	0.00	0.00	0.00	1.00	1.00	-8.35
hsa-miR-548k	0.00	0.00	0.00	1.00	1.00	-8.35
hsa-miR-3065-3p	0.00	0.00	0.00	1.00	1.00	-8.35
hsa-miR-548g-3p	0.00	0.00	0.00	1.00	1.00	-8.35
hsa-miR-448	0.00	0.00	0.00	1.00	1.00	-8.35
hsa-miR-3605-5p	0.00	0.00	0.00	1.00	1.00	-8.35
hsa-miR-510-3p	0.00	0.00	0.00	1.00	1.00	-8.35
hsa-miR-598-3p	-0.08	6.54	-0.16	0.88	1.00	-8.34
hsa-miR-876-5p	0.00	0.00	0.00	1.00	1.00	-8.35

hsa-miR-650	0.00	0.00	0.00	1.00	1.00	-8.35
hsa-miR-181d-3p	0.00	0.00	0.00	1.00	1.00	-8.35
hsa-miR-574-3p	-2.57	2.79	-1.16	0.32	1.00	-7.63
hsa-miR-652-5p	0.00	0.00	0.00	1.00	1.00	-8.35
hsa-miR-563	0.00	0.00	0.00	1.00	1.00	-8.35
hsa-miR-517c-3p+hsa-miR-519a-3p	0.00	0.00	0.00	1.00	1.00	-8.35
hsa-miR-660-3p	0.00	0.00	0.00	1.00	1.00	-8.35
hsa-miR-5010-3p	0.00	0.00	0.00	1.00	1.00	-8.35
hsa-miR-1302	0.00	0.00	0.00	1.00	1.00	-8.35
hsa-miR-642a-5p	0.00	0.00	0.00	1.00	1.00	-8.35
hsa-miR-502-5p	0.00	0.00	0.00	1.00	1.00	-8.35
hsa-miR-4647	0.00	0.00	0.00	1.00	1.00	-8.35
hsa-miR-184	0.00	0.00	0.00	1.00	1.00	-8.35
hsa-miR-200a-3p	-0.94	10.62	-2.55	0.07	1.00	-6.00
hsa-miR-301a-3p	-0.51	11.02	-1.11	0.34	1.00	-7.68
hsa-miR-4488	2.60	1.56	1.66	0.18	1.00	-7.05
hsa-miR-522-3p	0.00	0.00	0.00	1.00	1.00	-8.35
hsa-miR-487b-5p	0.00	0.00	0.00	1.00	1.00	-8.35
hsa-miR-5001-5p	0.00	0.00	0.00	1.00	1.00	-8.35
hsa-miR-802	0.00	0.00	0.00	1.00	1.00	-8.35
hsa-miR-378i	0.66	7.23	0.89	0.43	1.00	-7.89
hsa-miR-4286	-1.26	0.50	-1.44	0.23	1.00	-7.31
hsa-miR-532-3p	0.00	0.00	0.00	1.00	1.00	-8.35
hsa-miR-933	0.00	0.00	0.00	1.00	1.00	-8.35
hsa-miR-362-5p	-2.43	1.43	-1.17	0.32	1.00	-7.61
hsa-miR-4516	0.00	0.00	0.00	1.00	1.00	-8.35
hsa-miR-421	-0.99	5.91	-0.54	0.62	1.00	-8.17
hsa-miR-551b-3p	0.00	0.00	0.00	1.00	1.00	-8.35
hsa-miR-378f	0.00	0.00	0.00	1.00	1.00	-8.35
hsa-miR-543	0.00	0.00	0.00	1.00	1.00	-8.35

hsa-miR-30a-3p	0.00	0.00	0.00	1.00	1.00	-8.35
hsa-miR-324-3p	0.00	0.00	0.00	1.00	1.00	-8.35
hsa-miR-1305	0.00	0.00	0.00	1.00	1.00	-8.35
hsa-miR-584-3p	0.00	0.00	0.00	1.00	1.00	-8.35
hsa-miR-3147	0.00	0.00	0.00	1.00	1.00	-8.35
hsa-miR-548e-5p	0.00	0.00	0.00	1.00	1.00	-8.35
hsa-miR-607	0.00	0.00	0.00	1.00	1.00	-8.35
hsa-miR-1197	0.00	0.00	0.00	1.00	1.00	-8.35
hsa-miR-1285-5p	0.00	0.00	0.00	1.00	1.00	-8.35
hsa-miR-764	0.00	0.00	0.00	1.00	1.00	-8.35
hsa-miR-525-5p	0.00	0.00	0.00	1.00	1.00	-8.35
hsa-miR-1296-3p	0.00	0.00	0.00	1.00	1.00	-8.35
hsa-miR-4461	0.00	0.00	0.00	1.00	1.00	-8.35
hsa-miR-183-5p	-1.10	8.88	-2.47	0.08	1.00	-6.09
hsa-miR-615-3p	0.00	0.00	0.00	1.00	1.00	-8.35
hsa-miR-208b-3p	0.00	0.00	0.00	1.00	1.00	-8.35
hsa-miR-299-5p	0.00	0.00	0.00	1.00	1.00	-8.35
hsa-miR-1290	-1.73	0.69	-1.44	0.23	1.00	-7.31
hsa-miR-107	-0.22	9.89	-0.52	0.63	1.00	-8.19
hsa-miR-449b-5p	0.00	0.00	0.00	1.00	1.00	-8.35
hsa-miR-4707-5p	0.00	0.00	0.00	1.00	1.00	-8.35
hsa-miR-548y	0.00	0.00	0.00	1.00	1.00	-8.35
hsa-miR-370-3p	0.00	0.00	0.00	1.00	1.00	-8.35
hsa-miR-34a-5p	0.12	9.69	0.43	0.69	1.00	-8.24
hsa-miR-519c-3p	0.00	0.00	0.00	1.00	1.00	-8.35
hsa-miR-134-3p	0.00	0.00	0.00	1.00	1.00	-8.35
hsa-miR-2110	0.00	0.00	0.00	1.00	1.00	-8.35
hsa-miR-301a-5p	-2.14	0.85	-1.44	0.23	1.00	-7.31
hsa-miR-1973	0.00	0.00	0.00	1.00	1.00	-8.35
hsa-miR-643	0.00	0.00	0.00	1.00	1.00	-8.35

hsa-miR-328-5p	0.00	0.00	0.00	1.00	1.00	-8.35
hsa-miR-450a-2-3p	0.00	0.00	0.00	1.00	1.00	-8.35
hsa-miR-1272	0.00	0.00	0.00	1.00	1.00	-8.35
hsa-miR-199b-5p	-0.69	0.27	-1.44	0.23	1.00	-7.31
hsa-miR-210-3p	-1.73	0.69	-1.44	0.23	1.00	-7.31
hsa-let-7g-5p	-1.10	10.39	-3.20	0.04	1.00	-5.29
hsa-miR-26a-5p	0.17	8.95	0.35	0.75	1.00	-8.28
hsa-miR-937-3p	0.00	0.00	0.00	1.00	1.00	-8.35
hsa-miR-300	0.00	0.00	0.00	1.00	1.00	-8.35
hsa-miR-25-5p	0.00	0.00	0.00	1.00	1.00	-8.35
hsa-miR-888-5p	0.00	0.00	0.00	1.00	1.00	-8.35
hsa-miR-627-5p	0.00	0.00	0.00	1.00	1.00	-8.35
hsa-miR-186-5p	-2.31	0.92	-1.44	0.23	1.00	-7.31
hsa-miR-10b-5p	0.00	0.00	0.00	1.00	1.00	-8.35
hsa-miR-542-3p	0.00	0.00	0.00	1.00	1.00	-8.35
hsa-miR-887-5p	0.00	0.00	0.00	1.00	1.00	-8.35
hsa-miR-197-5p	0.00	0.00	0.00	1.00	1.00	-8.35
hsa-miR-939-5p	0.00	0.00	0.00	1.00	1.00	-8.35
hsa-miR-378g	0.84	3.23	0.29	0.79	1.00	-8.30
hsa-miR-95-3p	-1.34	6.12	-2.32	0.09	1.00	-6.27
hsa-miR-412-3p	0.00	0.00	0.00	1.00	1.00	-8.35
hsa-miR-1228-3p	0.00	0.00	0.00	1.00	1.00	-8.35
hsa-miR-301b-3p	0.00	0.00	0.00	1.00	1.00	-8.35
hsa-miR-27a-3p	0.00	0.00	0.00	1.00	1.00	-8.35
hsa-miR-1268b	0.00	0.00	0.00	1.00	1.00	-8.35
hsa-miR-514a-3p	0.00	0.00	0.00	1.00	1.00	-8.35
hsa-miR-548ar-3p	0.00	0.00	0.00	1.00	1.00	-8.35
hsa-miR-1255a	0.00	0.00	0.00	1.00	1.00	-8.35
hsa-miR-627-3p	0.00	0.00	0.00	1.00	1.00	-8.35
hsa-miR-1295a	0.00	0.00	0.00	1.00	1.00	-8.35

hsa-miR-597-5p	0.00	0.00	0.00	1.00	1.00	-8.35
hsa-miR-576-3p	0.00	0.00	0.00	1.00	1.00	-8.35
hsa-miR-891a-5p	0.00	0.00	0.00	1.00	1.00	-8.35
hsa-miR-133a-5p	0.00	0.00	0.00	1.00	1.00	-8.35
hsa-miR-301b-5p	0.00	0.00	0.00	1.00	1.00	-8.35
hsa-miR-210-5p	0.00	0.00	0.00	1.00	1.00	-8.35
hsa-miR-181a-2-3p	0.00	0.00	0.00	1.00	1.00	-8.35
hsa-miR-942-5p	-0.31	0.12	-1.44	0.23	1.00	-7.31
hsa-miR-133a-3p	0.00	0.00	0.00	1.00	1.00	-8.35
hsa-miR-23a-3p	-0.53	12.33	-1.12	0.33	1.00	-7.66
hsa-miR-548m	0.00	0.00	0.00	1.00	1.00	-8.35
hsa-miR-208a-3p	0.00	0.00	0.00	1.00	1.00	-8.35
hsa-miR-30e-5p	-1.01	6.70	-1.93	0.14	1.00	-6.73
hsa-miR-378d	0.00	0.00	0.00	1.00	1.00	-8.35
hsa-miR-335-5p	-2.14	0.85	-1.44	0.23	1.00	-7.31
hsa-miR-514b-5p	0.00	0.00	0.00	1.00	1.00	-8.35
hsa-miR-199a-3p+hsa-miR-199b-3p	-2.05	0.82	-1.44	0.23	1.00	-7.31
hsa-miR-378e	0.00	0.00	0.00	1.00	1.00	-8.35
hsa-miR-4485-3p	0.00	0.00	0.00	1.00	1.00	-8.35
hsa-miR-1537-3p	0.00	0.00	0.00	1.00	1.00	-8.35
hsa-miR-548v	0.00	0.00	0.00	1.00	1.00	-8.35
hsa-miR-487a-3p	0.00	0.00	0.00	1.00	1.00	-8.35
hsa-miR-3613-3p	0.00	0.00	0.00	1.00	1.00	-8.35
hsa-miR-1245a	0.00	0.00	0.00	1.00	1.00	-8.35
hsa-miR-498	0.00	0.00	0.00	1.00	1.00	-8.35
hsa-miR-495-5p	0.00	0.00	0.00	1.00	1.00	-8.35
hsa-miR-3614-5p	0.00	0.00	0.00	1.00	1.00	-8.35
hsa-miR-137	0.00	0.00	0.00	1.00	1.00	-8.35
hsa-miR-187-3p	0.85	0.51	0.83	0.46	1.00	-7.95
hsa-miR-1249-3p	0.00	0.00	0.00	1.00	1.00	-8.35

hsa-miR-3168	0.00	0.00	0.00	1.00	1.00	-8.35
hsa-miR-664a-3p	-1.10	2.02	-0.41	0.70	1.00	-8.25
hsa-miR-639	0.00	0.00	0.00	1.00	1.00	-8.35
hsa-miR-337-3p	0.00	0.00	0.00	1.00	1.00	-8.35
hsa-miR-363-3p	0.81	5.76	1.24	0.29	1.00	-7.54
hsa-miR-1185-5p	0.00	0.00	0.00	1.00	1.00	-8.35
hsa-miR-767-5p	0.00	0.00	0.00	1.00	1.00	-8.35
hsa-miR-1283	0.00	0.00	0.00	1.00	1.00	-8.35
hsa-miR-199a-5p	0.00	0.00	0.00	1.00	1.00	-8.35
hsa-miR-16-5p	-0.99	12.15	-2.53	0.07	1.00	-6.03
hsa-miR-299-3p	0.00	0.00	0.00	1.00	1.00	-8.35
hsa-miR-1976	0.00	0.00	0.00	1.00	1.00	-8.35
hsa-miR-454-3p	-1.07	9.27	-2.87	0.05	1.00	-5.64
hsa-miR-345-3p	0.00	0.00	0.00	1.00	1.00	-8.35
hsa-miR-556-5p	0.00	0.00	0.00	1.00	1.00	-8.35
hsa-miR-487b-3p	0.00	0.00	0.00	1.00	1.00	-8.35
hsa-miR-513a-3p	0.00	0.00	0.00	1.00	1.00	-8.35
hsa-miR-885-5p	0.00	0.00	0.00	1.00	1.00	-8.35
hsa-miR-521	0.00	0.00	0.00	1.00	1.00	-8.35
hsa-miR-147b	0.00	0.00	0.00	1.00	1.00	-8.35
hsa-miR-2113	0.00	0.00	0.00	1.00	1.00	-8.35
hsa-miR-200b-3p	-0.62	12.66	-1.47	0.23	1.00	-7.27
hsa-miR-18b-5p	0.00	0.00	0.00	1.00	1.00	-8.35
hsa-miR-188-3p	0.00	0.00	0.00	1.00	1.00	-8.35
hsa-miR-6720-3p	0.00	0.00	0.00	1.00	1.00	-8.35
hsa-miR-582-3p	0.00	0.00	0.00	1.00	1.00	-8.35
hsa-miR-1200	0.00	0.00	0.00	1.00	1.00	-8.35
hsa-miR-378h	0.00	0.00	0.00	1.00	1.00	-8.35
hsa-miR-4792	0.00	0.00	0.00	1.00	1.00	-8.35
hsa-miR-572	0.00	0.00	0.00	1.00	1.00	-8.35

hsa-miR-660-5p	-3.82	4.48	-1.99	0.13	1.00	-6.66
hsa-miR-449a	0.00	0.00	0.00	1.00	1.00	-8.35
hsa-miR-302a-3p	0.00	0.00	0.00	1.00	1.00	-8.35
hsa-miR-27b-3p	1.04	10.32	2.31	0.09	1.00	-6.27
hsa-miR-1281	0.00	0.00	0.00	1.00	1.00	-8.35
hsa-miR-106b-5p	-1.00	9.88	-2.20	0.10	1.00	-6.41
hsa-miR-182-3p	0.00	0.00	0.00	1.00	1.00	-8.35
hsa-miR-455-3p	0.00	0.00	0.00	1.00	1.00	-8.35
hsa-miR-128-3p	0.00	0.00	0.00	1.00	1.00	-8.35
hsa-miR-376c-5p	0.00	0.00	0.00	1.00	1.00	-8.35
hsa-miR-1275	0.00	0.00	0.00	1.00	1.00	-8.35
hsa-miR-1224-3p	0.00	0.00	0.00	1.00	1.00	-8.35
hsa-miR-518e-3p	0.00	0.00	0.00	1.00	1.00	-8.35
hsa-miR-625-5p	0.15	4.22	0.08	0.94	1.00	-8.35
hsa-miR-302a-5p	0.00	0.00	0.00	1.00	1.00	-8.35
hsa-miR-130a-3p	0.00	0.00	0.00	1.00	1.00	-8.35
hsa-miR-136-5p	0.00	0.00	0.00	1.00	1.00	-8.35
hsa-miR-758-5p	0.00	0.00	0.00	1.00	1.00	-8.35
hsa-miR-1277-3p	0.00	0.00	0.00	1.00	1.00	-8.35
hsa-miR-4425	0.00	0.00	0.00	1.00	1.00	-8.35
hsa-miR-511-5p	0.00	0.00	0.00	1.00	1.00	-8.35
hsa-miR-154-5p	0.00	0.00	0.00	1.00	1.00	-8.35
hsa-miR-934	0.00	0.00	0.00	1.00	1.00	-8.35
hsa-miR-330-3p	0.00	0.00	0.00	1.00	1.00	-8.35
hsa-miR-222-3p	4.25	4.56	2.49	0.08	1.00	-6.07
hsa-miR-4741	0.00	0.00	0.00	1.00	1.00	-8.35
hsa-miR-513c-3p	0.00	0.00	0.00	1.00	1.00	-8.35
hsa-miR-330-5p	0.00	0.00	0.00	1.00	1.00	-8.35
hsa-miR-760	0.00	0.00	0.00	1.00	1.00	-8.35
hsa-miR-25-3p	-0.65	12.58	-1.33	0.26	1.00	-7.43

hsa-miR-410-3p	0.00	0.00	0.00	1.00	1.00	-8.35
hsa-miR-376b-3p	0.00	0.00	0.00	1.00	1.00	-8.35
hsa-miR-484	-0.93	0.37	-1.44	0.23	1.00	-7.31
hsa-miR-539-3p	0.00	0.00	0.00	1.00	1.00	-8.35
hsa-miR-548c-5p+hsa-miR-548o-5p+hsa-miR-548am-5p	0.00	0.00	0.00	1.00	1.00	-8.35
hsa-miR-649	0.00	0.00	0.00	1.00	1.00	-8.35
hsa-miR-631	0.00	0.00	0.00	1.00	1.00	-8.35
hsa-miR-3185	0.00	0.00	0.00	1.00	1.00	-8.35
hsa-miR-1909-3p	0.00	0.00	0.00	1.00	1.00	-8.35
hsa-miR-1249-5p	0.00	0.00	0.00	1.00	1.00	-8.35
hsa-miR-129-5p	0.00	0.00	0.00	1.00	1.00	-8.35
hsa-miR-3615	0.00	0.00	0.00	1.00	1.00	-8.35
hsa-miR-1254	0.00	0.00	0.00	1.00	1.00	-8.35
hsa-miR-296-3p	0.00	0.00	0.00	1.00	1.00	-8.35
hsa-miR-323a-3p	0.00	0.00	0.00	1.00	1.00	-8.35
hsa-miR-876-3p	0.00	0.00	0.00	1.00	1.00	-8.35
hsa-miR-769-5p	-0.51	6.10	-0.89	0.43	1.00	-7.90
hsa-miR-146a-5p	0.00	0.00	0.00	1.00	1.00	-8.35
hsa-let-7e-5p	0.39	10.55	1.39	0.25	1.00	-7.36
hsa-miR-376c-3p	0.00	0.00	0.00	1.00	1.00	-8.35
hsa-miR-361-3p	-2.20	2.64	-0.86	0.45	1.00	-7.93
hsa-miR-99a-5p	1.08	8.10	1.83	0.15	1.00	-6.84
hsa-miR-485-3p	0.00	0.00	0.00	1.00	1.00	-8.35
hsa-miR-1322	0.00	0.00	0.00	1.00	1.00	-8.35
hsa-miR-1260b	0.00	0.00	0.00	1.00	1.00	-8.35
hsa-miR-507	0.00	0.00	0.00	1.00	1.00	-8.35
hsa-miR-642a-3p	0.00	0.00	0.00	1.00	1.00	-8.35
hsa-miR-378b	0.00	0.00	0.00	1.00	1.00	-8.35
hsa-miR-331-5p	0.00	0.00	0.00	1.00	1.00	-8.35

hsa-miR-3613-5p	0.00	0.00	0.00	1.00	1.00	-8.35
hsa-miR-2116-5p	0.00	0.00	0.00	1.00	1.00	-8.35
hsa-miR-204-5p	0.00	0.00	0.00	1.00	1.00	-8.35
hsa-miR-22-3p	-0.03	8.03	-0.07	0.94	1.00	-8.35
hsa-miR-323a-5p	0.00	0.00	0.00	1.00	1.00	-8.35
hsa-miR-6511a-3p	0.00	0.00	0.00	1.00	1.00	-8.35
hsa-miR-554	0.00	0.00	0.00	1.00	1.00	-8.35
hsa-miR-600	0.00	0.00	0.00	1.00	1.00	-8.35
hsa-miR-1303	0.00	0.00	0.00	1.00	1.00	-8.35
hsa-miR-517b-3p	0.00	0.00	0.00	1.00	1.00	-8.35
hsa-miR-106a-5p+hsa-miR-17-5p	-0.08	7.60	-0.11	0.92	1.00	-8.35
hsa-miR-496	0.00	0.00	0.00	1.00	1.00	-8.35
hsa-miR-3190-3p	0.00	0.00	0.00	1.00	1.00	-8.35
hsa-miR-148b-3p	-0.91	9.28	-2.05	0.12	1.00	-6.58
hsa-miR-223-3p	0.00	0.00	0.00	1.00	1.00	-8.35
hsa-miR-196b-5p	-2.42	0.97	-1.44	0.23	1.00	-7.31
hsa-miR-652-3p	-4.11	3.16	-2.21	0.10	1.00	-6.40
hsa-miR-637	0.00	0.00	0.00	1.00	1.00	-8.35
hsa-miR-5001-3p	0.00	0.00	0.00	1.00	1.00	-8.35
hsa-miR-3130-3p	0.00	0.00	0.00	1.00	1.00	-8.35
hsa-miR-630	0.00	0.00	0.00	1.00	1.00	-8.35
hsa-miR-197-3p	-2.47	2.04	-1.93	0.14	1.00	-6.72
hsa-miR-1245b-5p	0.00	0.00	0.00	1.00	1.00	-8.35
hsa-miR-648	0.00	0.00	0.00	1.00	1.00	-8.35
hsa-miR-552-3p	0.00	0.00	0.00	1.00	1.00	-8.35
hsa-miR-346	0.00	0.00	0.00	1.00	1.00	-8.35
hsa-miR-377-3p	0.00	0.00	0.00	1.00	1.00	-8.35
hsa-miR-219b-3p	0.00	0.00	0.00	1.00	1.00	-8.35
hsa-miR-744-5p	0.00	0.00	0.00	1.00	1.00	-8.35
hsa-miR-224-5p	0.00	0.00	0.00	1.00	1.00	-8.35

hsa-miR-1306-3p	0.00	0.00	0.00	1.00	1.00	-8.35
hsa-miR-495-3p	0.00	0.00	0.00	1.00	1.00	-8.35
hsa-miR-3127-5p	0.00	0.00	0.00	1.00	1.00	-8.35
hsa-miR-1297	0.00	0.00	0.00	1.00	1.00	-8.35
hsa-miR-664b-3p	0.00	0.00	0.00	1.00	1.00	-8.35
hsa-miR-1270	0.00	0.00	0.00	1.00	1.00	-8.35
hsa-miR-514b-3p	0.00	0.00	0.00	1.00	1.00	-8.35
hsa-miR-198	0.00	0.00	0.00	1.00	1.00	-8.35
hsa-miR-146b-5p	0.00	0.00	0.00	1.00	1.00	-8.35
hsa-miR-298	0.00	0.00	0.00	1.00	1.00	-8.35
hsa-miR-936	0.00	0.00	0.00	1.00	1.00	-8.35
hsa-miR-205-5p	4.04	4.08	2.26	0.10	1.00	-6.34
hsa-miR-520h	0.00	0.00	0.00	1.00	1.00	-8.35
hsa-miR-506-5p	0.00	0.00	0.00	1.00	1.00	-8.35
hsa-miR-3131	0.00	0.00	0.00	1.00	1.00	-8.35
hsa-miR-432-5p	0.00	0.00	0.00	1.00	1.00	-8.35
hsa-miR-548j-5p	0.00	0.00	0.00	1.00	1.00	-8.35
hsa-miR-1271-5p	0.00	0.00	0.00	1.00	1.00	-8.35
hsa-miR-4707-3p	0.00	0.00	0.00	1.00	1.00	-8.35
hsa-miR-548z+hsa-miR-548h-3p	0.00	0.00	0.00	1.00	1.00	-8.35
hsa-miR-485-5p	0.00	0.00	0.00	1.00	1.00	-8.35
hsa-miR-331-3p	-0.49	6.11	-1.47	0.23	1.00	-7.27
hsa-miR-33a-5p	-2.54	1.02	-1.44	0.23	1.00	-7.31
hsa-miR-502-3p	0.00	0.00	0.00	1.00	1.00	-8.35
hsa-miR-605-5p	0.00	0.00	0.00	1.00	1.00	-8.35
hsa-miR-1178-3p	0.00	0.00	0.00	1.00	1.00	-8.35
hsa-miR-765	0.00	0.00	0.00	1.00	1.00	-8.35
hsa-miR-517a-3p	0.00	0.00	0.00	1.00	1.00	-8.35
hsa-miR-3151-5p	0.00	0.00	0.00	1.00	1.00	-8.35
hsa-miR-661	0.00	0.00	0.00	1.00	1.00	-8.35

hsa-miR-1185-1-3p	0.00	0.00	0.00	1.00	1.00	-8.35
hsa-miR-573	0.00	0.00	0.00	1.00	1.00	-8.35
hsa-miR-486-3p	0.00	0.00	0.00	1.00	1.00	-8.35
hsa-miR-382-3p	0.00	0.00	0.00	1.00	1.00	-8.35
hsa-miR-566	0.00	0.00	0.00	1.00	1.00	-8.35
hsa-miR-508-3p	0.00	0.00	0.00	1.00	1.00	-8.35
hsa-miR-411-5p	0.00	0.00	0.00	1.00	1.00	-8.35
hsa-miR-433-3p	0.00	0.00	0.00	1.00	1.00	-8.35
hsa-miR-590-3p	0.00	0.00	0.00	1.00	1.00	-8.35
hsa-miR-663a	0.00	0.00	0.00	1.00	1.00	-8.35
hsa-miR-638	0.00	0.00	0.00	1.00	1.00	-8.35
hsa-miR-4455	0.00	0.00	0.00	1.00	1.00	-8.35
hsa-let-7f-5p	-1.30	9.43	-2.94	0.05	1.00	-5.57
hsa-miR-1-5p	0.00	0.00	0.00	1.00	1.00	-8.35
hsa-miR-766-3p	0.00	0.00	0.00	1.00	1.00	-8.35
hsa-miR-641	0.00	0.00	0.00	1.00	1.00	-8.35
hsa-miR-211-3p	0.00	0.00	0.00	1.00	1.00	-8.35
hsa-miR-323b-5p	0.00	0.00	0.00	1.00	1.00	-8.35
hsa-miR-92a-1-5p	0.00	0.00	0.00	1.00	1.00	-8.35
hsa-miR-548ad-3p	0.00	0.00	0.00	1.00	1.00	-8.35
hsa-miR-562	0.00	0.00	0.00	1.00	1.00	-8.35
hsa-miR-296-5p	-0.12	9.56	-2.48	0.08	1.00	-6.08
hsa-miR-548ak	0.00	0.00	0.00	1.00	1.00	-8.35
hsa-miR-544a	0.00	0.00	0.00	1.00	1.00	-8.35
hsa-miR-361-5p	0.32	8.32	0.79	0.48	1.00	-7.99
hsa-miR-181c-5p	2.76	1.65	1.65	0.19	1.00	-7.06
hsa-miR-203a-3p	-1.42	8.81	-2.36	0.09	1.00	-6.22
hsa-miR-614	0.00	0.00	0.00	1.00	1.00	-8.35
hsa-miR-513c-5p	0.00	0.00	0.00	1.00	1.00	-8.35
hsa-miR-520e	0.00	0.00	0.00	1.00	1.00	-8.35

hsa-miR-1252-5p	0.00	0.00	0.00	1.00	1.00	-8.35
hsa-miR-4448	0.00	0.00	0.00	1.00	1.00	-8.35
hsa-miR-892b	0.00	0.00	0.00	1.00	1.00	-8.35
hsa-miR-874-3p	0.00	0.00	0.00	1.00	1.00	-8.35
hsa-miR-134-5p+hsa-miR-6728-5p	0.00	0.00	0.00	1.00	1.00	-8.35
hsa-miR-4431	0.00	0.00	0.00	1.00	1.00	-8.35
hsa-miR-761	0.00	0.00	0.00	1.00	1.00	-8.35
hsa-miR-6511a-5p	0.00	0.00	0.00	1.00	1.00	-8.35
hsa-miR-1304-3p	0.00	0.00	0.00	1.00	1.00	-8.35
hsa-miR-128-2-5p	0.00	0.00	0.00	1.00	1.00	-8.35
hsa-miR-30d-5p	-0.31	9.44	-0.52	0.63	1.00	-8.19
hsa-miR-378c	0.00	0.00	0.00	1.00	1.00	-8.35
hsa-miR-655-3p	0.00	0.00	0.00	1.00	1.00	-8.35
hsa-miR-99b-5p	0.58	10.36	1.16	0.32	1.00	-7.62
hsa-miR-4532	0.00	0.00	0.00	1.00	1.00	-8.35
hsa-miR-206	0.00	0.00	0.00	1.00	1.00	-8.35
hsa-miR-1269b	0.00	0.00	0.00	1.00	1.00	-8.35
hsa-miR-510-5p	0.00	0.00	0.00	1.00	1.00	-8.35
hsa-miR-628-5p	0.00	0.00	0.00	1.00	1.00	-8.35
hsa-miR-2278	0.00	0.00	0.00	1.00	1.00	-8.35
hsa-miR-1296-5p	0.00	0.00	0.00	1.00	1.00	-8.35
hsa-miR-664b-5p	0.00	0.00	0.00	1.00	1.00	-8.35
hsa-miR-483-5p	0.00	0.00	0.00	1.00	1.00	-8.35
hsa-miR-125a-5p	0.42	11.35	1.59	0.20	1.00	-7.13
hsa-miR-3140-5p	0.00	0.00	0.00	1.00	1.00	-8.35
hsa-miR-3916	0.00	0.00	0.00	1.00	1.00	-8.35
hsa-miR-1255b-5p	0.00	0.00	0.00	1.00	1.00	-8.35
hsa-miR-651-3p	0.00	0.00	0.00	1.00	1.00	-8.35
hsa-miR-629-5p	-0.31	0.12	-1.44	0.23	1.00	-7.31
hsa-miR-3065-5p	2.33	1.40	1.65	0.19	1.00	-7.07

hsa-miR-520b	0.00	0.00	0.00	1.00	1.00	-8.35
hsa-miR-3605-3p	0.00	0.00	0.00	1.00	1.00	-8.35
hsa-miR-548d-3p	0.00	0.00	0.00	1.00	1.00	-8.35
hsa-miR-494-5p	0.00	0.00	0.00	1.00	1.00	-8.35
hsa-miR-624-3p	0.00	0.00	0.00	1.00	1.00	-8.35
hsa-miR-221-5p	0.00	0.00	0.00	1.00	1.00	-8.35
hsa-miR-520a-3p	0.00	0.00	0.00	1.00	1.00	-8.35
hsa-miR-924	0.00	0.00	0.00	1.00	1.00	-8.35
hsa-miR-512-3p	0.00	0.00	0.00	1.00	1.00	-8.35
hsa-miR-766-5p	0.00	0.00	0.00	1.00	1.00	-8.35
hsa-miR-548h-5p	0.00	0.00	0.00	1.00	1.00	-8.35
hsa-miR-297	0.00	0.00	0.00	1.00	1.00	-8.35
hsa-miR-30a-5p	-0.32	3.01	-0.11	0.92	1.00	-8.35
hsa-miR-362-3p	-2.47	1.23	-1.31	0.27	1.00	-7.46
hsa-miR-548q	0.00	0.00	0.00	1.00	1.00	-8.35
hsa-miR-146b-3p	0.00	0.00	0.00	1.00	1.00	-8.35
hsa-miR-182-5p	-1.29	7.43	-2.97	0.05	1.00	-5.54
hsa-miR-1301-3p	0.00	0.00	0.00	1.00	1.00	-8.35
hsa-miR-494-3p	0.00	0.00	0.00	1.00	1.00	-8.35
hsa-miR-452-5p	0.00	0.00	0.00	1.00	1.00	-8.35
hsa-miR-599	0.00	0.00	0.00	1.00	1.00	-8.35
hsa-miR-1286	0.00	0.00	0.00	1.00	1.00	-8.35
hsa-miR-501-3p	0.00	0.00	0.00	1.00	1.00	-8.35
hsa-miR-1915-3p	0.00	0.00	0.00	1.00	1.00	-8.35
hsa-miR-409-5p	0.00	0.00	0.00	1.00	1.00	-8.35
