# Supplementary material for: Relationship of micro-RNA, mRNA and eIF Expression in Tamoxifen-Adapted MCF-7 Breast Cancer Cells: Impact of miR-1972 on Gene Expression, Proliferation and Migration
Source: Biomolecules. 2022 Jun 29;12(7):916. doi: 10.3390/biom12070916 (PMC9312698; doi:10.3390/biom12070916)
Supplement: Supplementary file 1 [file biomolecules-12-00916-s001.zip › Table S2 miRNA statistic.pdf]

Table S2A. Relative expression of micro RNAs as determined by qRT PCR. Values are expressed as $\log_2$Fc relative to RPL-13 and MCF-7.

	MCF-7	Tam	MDA-MB-468	HS578T	SkBr3	MDA-MB-231	UACC3199	T47D
miR-1972	-0.02	-2.63	-5.82	-5.17	-7.22	-6.69	-4.73	-5.16
miR-455	-0.11	2.88	b.d.*	3.55	4.00	2.92	4.59	1.21
miR-213	-0.06	2.25	1.89	2.64	1.42	3.29	3.24	1.86
miR-375	-0.14	-4.90	-1.52	-12.20	-1.45	-11.64	-0.76	0.85
miR-181b-5p	-0.08	4.89	6.97	5.99	5.10	6.33	5.16	7.57
miR-181d-5p	-0.02	5.30	5.78	5.96	6.29	7.47	4.83	7.54

* below detection limit

Table S2B. Statistical analysis of miRNA expression in breast cancer cell lines. p-values of ANOVA post-hoc analysis are shown. Either LSD or Tamhane T2 post hoc analysis was applied, depending on results of the Levene test for equal variances.

miR1972 (LSD)	MCF-7	TamR	MDA-MB-468	HS578T	Sk-Br3	MDA-MB-231	UACC3199	T47D
MCF-7		< 0.001		< 0.001	< 0.001	< 0.001	< 0.001	0.001
TamR	< 0.001			0.077	0.006	0.906	< 0.001	< 0.001
MDA-MB-468	< 0.001	< 0.001						
HS578T	< 0.001	< 0.001	0.173		0.258	0.118	0.015	< 0.001
SkBr3	< 0.001	< 0.001	0.014	< 0.001		0.012	0.148	< 0.001
MDA-MB-231	< 0.001	< 0.001	0.108	0.008	0.360		< 0.001	< 0.001
UACC3199	< 0.001	< 0.001	0.029	0.356	< 0.001	0.001		< 0.001
T47D	< 0.001	< 0.001	0.169	0.989	0.001	0.008	0.363	miR455 (LSD)

miR-213 (T2)	MCF-7	TamR	MDA-MB468	HS578T	SkBr3	MDA-MB231	UACC3199	T47D
MCF-7		< 0.001	0.869	0.722	0.292	0.049	0.932	0.473
TamR	0.003		0.176	0.907	0.007	0.196	< 0.001	< 0.001
MDA-MB-468	1.000	1.000		0.625	1.000	0.026	0.999	0.475
HS578T	0.001	0.999	1.000		0.779	1.000	0.794	0.734
SkBr3	0.014	0.555	1.000	0.144		0.075	0.944	0.069
MDA-MB-231	0.115	0.935	1.000	0.999	0.543		0.093	0.062
UACC3199	< 0.001	0.394	1.000	0.864	0.041	1.000		0.099
T47D	0.534	1.000	1.000	0.997	1.000	0.845	0.838	miR-375 (T2)

miR-181b-5p (LSD)	MCF-7	TamR	MDA-MB-468	HS578T	Sk-Br3	MDA-MB-231	UACC3199	T47D
MCF-7		< 0.001	< 0.001	< 0.001	< 0.001	< 0.001	< 0.001	< 0.001
TamR	< 0.001		0.371	0.227	0.114	0.001	0.392	0.001
MDA-MB-468	< 0.001	0.008		0.758	0.429	0.008	0.111	0.006
HS578T	< 0.001	0.133	0.203		0.603	0.016	0.063	0.012
Sk-Br3	< 0.001	0.768	0.022	0.249		0.079	0.034	0.065
MDA-MB-231	< 0.001	0.053	0.403	0.648	0.115		< 0.001	0.912
UACC3199	< 0.001	0.698	0.026	0.285	0.930	0.135		< 0.001
T47D	< 0.001	0.001	0.437	0.049	0.004	0.116	0.005	miR-181d-5p (LSD)

Significance:	p< 0.001	p< 0.05	p< 0.1
