# Supplementary material for: Relationship of micro-RNA, mRNA and eIF Expression in Tamoxifen-Adapted MCF-7 Breast Cancer Cells: Impact of miR-1972 on Gene Expression, Proliferation and Migration
Source: Biomolecules. 2022 Jun 29;12(7):916. doi: 10.3390/biom12070916 (PMC9312698; doi:10.3390/biom12070916)
Supplement: Supplementary file 1 [file biomolecules-12-00916-s001.zip › Table S3 Pathway enrichment results.pdf]

Table S3 A. Pathway enrichment analysis for tamoxifen regulated miRNAs.

Predicted target genes of the tamoxifen regulated miRNAs that were also regulated by tamoxifen adaption were pooled and submitted for pathway enrichment analysis to the EnrichR website. Results are shown for $p_{adj} < 0.05$.

KEGG pathway enrichment

Term	Overlap	P-value	Adjusted P-value	Odds Ratio	Combined Score	Genes
Leishmaniasis	6/77	2.31E-04	0.042510861	7.603318541	63.66073308	C3;NFKBIA;HLA-DRB5;HLA-DPB1;FOS;HLA-DRB1
Morphine addiction	6/91	5.70E-04	0.042510861	6.346494762	47.40443199	GABBR2;PDE4D;ADORA1;PDE8B;ADCY5;KCNJ3
Th1 and Th2 cell differentiation	6/92	6.04E-04	0.042510861	6.272379739	46.48612117	NFKBIA;HLA-DRB5;JAG1;HLA-DPB1;FOS;HLA-DRB1
Human T-cell leukemia virus 1 infection	9/219	8.66E-04	0.045696296	3.881944444	27.37276189	NFKBIA;EGR1;NRP1;HLA-DRB5;MAP3K1;HLA-DPB1;FOS;HLA-DRB1;ADCY5

GEO kinase perturbations up

Term	Overlap	P-value	Adjusted P-value	Odds Ratio	Combined Score	Genes
LRRK2 activemutant 159 GSE36321	17/300	5.57E-08	7.61E-06	5.629315031	94.02315985	MAP3K1;LIMCH1;LUM;PCDH7;SAMD4A;LAMC1;NR3C1;SYNE1;SLC7A5;RAB31;DRAM1;KLF5;PLAU;SYNDIG1;CPE;STXBP6;TNS3
LRRK2 mutant 33 GDS4401	17/300	5.57E-08	7.61E-06	5.629315031	94.02315985	MAP3K1;LIMCH1;LUM;PCDH7;SAMD4A;LAMC1;NR3C1;SYNE1;SLC7A5;RAB31;DRAM1;KLF5;PLAU;SYNDIG1;CPE;STXBP6;TNS3
FGFR3 druginhibition 36 GDS5023	16/300	3.15E-07	2.15E-05	5.253992857	78.64961128	SEMA5A;AHNAK;IGFBP5;PCDH7;RIPK4;TNFAIP2;PTK6;LAMC1;NFKBIA;AR;TOM1L2;RCAN1;PPP1R3B;PACS1;ERBB4;ST3GAL1
BRAF overexpression 180 GSE46801	16/300	3.15E-07	2.15E-05	5.253992857	78.64961128	EGR1;NRP1;SLC14A1;B4GALT1;KCNK10;FAM46C;TNFRSF11B;INHBA;NR3C1;C3;RAB31;S100A16;PODXL;CPE;TGFBI;RGL1
CDK19 knockdown 162 GSE38061	12/300	1.59E-04	0.006200996	3.812010955	33.34216183	TOM1L2;ALDH1A3;TCF7L1;SYT1;PODXL;OSBPL3;SCNN1A;PDK4;SPRY1;TNS4;CDC42BPA;ZFP36L1
CDK8 knockdown 163 GSE38061	12/300	1.59E-04	0.006200996	3.812010955	33.34216183	NFKBIA;TOM1L2;ALDH1A3;JAG1;PLAU;TESC;SUSD1;GRHL3;ANTXR2;TNS4;BLVRA;ZFP36L1
ROCK2 knockdown 157 GSE34769	12/300	1.59E-04	0.006200996	3.812010955	33.34216183	C3;NFKBIA;SGCE;EGR1;PLAU;PDK4;ANKS6;IRF8;CHAC1;FOS;PARP8;ZFP36L1
CDK8 knockdown 161 GSE38061	11/300	6.18E-04	0.018745182	3.465802154	25.60903989	NFKBIA;TOM1L2;JAG1;KLF5;AHNAK;TNFAIP2;AK4;TNS4;CDC42BPA;HS3ST1;ZFP36L1
ERBB3 drugactivation 70 GSE21463	11/300	6.18E-04	0.018745182	3.465802154	25.60903989	DOCK10;SGCE;PREX1;TOM1L2;EHD3;LIMCH1;PSD3;CPE;LDLRAD4;CDC42BPA;SYNE1
ULK1 knockout 198 GSE60778	10/300	0.002195838	0.046112597	3.125100241	19.12933717	ST6GAL1;TCF7L1;AHNAK;SLC17A5;SAMD4A;COL4A6;SMS;CDC42BPA;KAZALD1;DCLK1
ULK1 knockout 197 GSE60778	10/300	0.002195838	0.046112597	3.125100241	19.12933717	SOCS2;ST6GAL1;TCF7L1;LURAP1L;MAP2;SALL4;ANG;SULF1;CDC42BPA;JDP2
SNRK overexpression 127 GSE30185	10/300	0.002195838	0.046112597	3.125100241	19.12933717	GABBR2;ALDH1A3;RAB31;CXCL12;TPK1;RAB15;GFRA1;MBP;MAPK4;UGT1A6
HIPK2 defectivemutant 29 GDS4233	10/300	0.002195838	0.046112597	3.125100241	19.12933717	DOCK10;NRP1;BACE2;DEPTOR;IGFBP5;PCDH10;FAM46C;FAXDC2;GPR65;PCDH7

GEO kinase perturbations down

Term	Overlap	P-value	Adjusted P-value	Odds Ratio	Combined Score	Genes
PLK1 druginhibition 181 GSE46856	14/300	8.22E-06	0.002220049	4.521360156	52.93901535	MESP1;IGFBP5;TESC;C15ORF52;UBE2QL1;KAZALD1;C6ORF211;PAK1;ZNF618;TRIM29;RAB15;PLEKHO1;TNS4;SAMD12
EGFR drugactivation 30 GDS4361	12/300	1.59E-04	0.021464985	3.812010955	33.34216183	C3;ALDH1A3;ST6GAL1;B4GALT1;SH3PXD2A;PPP1R3B;PLAU;RIPK4;TNFRSF11B;FOS;TNS3;ZFP36L1

Table S3 B

Pathhway enrichment analysis using the GEO kinase up/down perturbations associated with the Tamoxifen regulated miRNAs

The list of 15 kinases was analyzed by the EnrichR website. An adjusted p-value of less than 0.01 was required to appear in this list.

Kinases used for this analysis:

BRAF	CDK19	CDK8	DDR1	EGFR	ERBB3	SNRK	
FGFR3	HIPK2	JAK2	LRRK2	PLK1	ROCK2	SYK	ULK1

KEGG 2021

Term	Overlap	P-value	Adjusted P-value	Odds Ratio	Combined Score	Genes
Bladder cancer	3/41	3.58E-06	2.41E-04	1.31E+02	1645.78	BRAF;FGFR3;EGFR
PI3K-Akt signaling pathway	5/354	4.38E-06	2.41E-04	2.81E+01	347.08	SYK;ERBB3;JAK2;FGFR3;EGFR
Proteoglycans in cancer	4/205	1.34E-05	4.69E-04	3.58E+01	401.62	ERBB3;ROCK2;BRAF;EGFR
Regulation of actin cytoskeleton	4/218	1.71E-05	4.69E-04	3.36E+01	368.84	ROCK2;BRAF;FGFR3;EGFR
Pathways in cancer	5/531	3.12E-05	5.96E-04	1.85E+01	191.92	ROCK2;BRAF;JAK2;FGFR3;EGFR
ErbB signaling pathway	3/85	3.25E-05	5.96E-04	6.07E+01	627.11	ERBB3;BRAF;EGFR
MAPK signaling pathway	4/294	5.50E-05	8.64E-04	2.47E+01	242.24	ERBB3;BRAF;FGFR3;EGFR
FoxO signaling pathway	3/131	1.18E-04	1.62E-03	3.88E+01	350.80	PLK1;BRAF;EGFR
Chemokine signaling pathway	3/192	3.64E-04	4.45E-03	2.62E+01	207.35	ROCK2;BRAF;JAK2
Focal adhesion	3/201	4.16E-04	4.58E-03	2.50E+01	194.48	ROCK2;BRAF;EGFR
Rap1 signaling pathway	3/210	4.73E-04	4.73E-03	2.39E+01	182.88	BRAF;FGFR3;EGFR
Calcium signaling pathway	3/240	6.98E-04	6.40E-03	2.08E+01	151.39	ERBB3;FGFR3;EGFR
Endometrial cancer	2/58	8.47E-04	7.17E-03	5.48E+01	387.29	BRAF;EGFR
Central carbon metabolism in cancer	2/70	1.23E-03	8.46E-03	4.51E+01	301.90	FGFR3;EGFR
Melanoma	2/72	1.30E-03	8.46E-03	4.38E+01	290.80	BRAF;EGFR
Non-small cell lung cancer	2/72	1.30E-03	8.46E-03	4.38E+01	290.80	BRAF;EGFR
Glioma	2/75	1.41E-03	8.46E-03	4.20E+01	275.41	BRAF;EGFR
Pancreatic cancer	2/76	1.45E-03	8.46E-03	4.14E+01	270.59	BRAF;EGFR
MicroRNAs in cancer	3/310	1.46E-03	8.46E-03	1.60E+01	104.61	ERBB3;FGFR3;EGFR

Reactome 2016

Term	Overlap	P-value	Adjusted P-value	Odds Ratio	Combined Score	Genes
Signaling by SCF-KIT Homo sapiens R-HSA-1433557	16/325	5.02E-20	0.000	53.56770567	2380.48879	SRC;INSR;BRAF;PIK3R1;EGFR;FER;ERBB3;ERBB4;FES;ERBB2;JAK2;JAK3;FGFR3;FGFR2;JAK1;FGFR1
Interleukin-3. 5 and GM-CSF signaling Homo sapiens R-HSA-512988	15/261	9.31E-20	0.000	60.11890244	2634.46995	SYK;SRC;BRAF;PIK3R1;EGFR;HCK;ERBB3;ERBB4;ERBB2;JAK2;JAK3;FGFR3;FGFR2;JAK1;FGFR1
Signaling by Interleukins Homo sapiens R-HSA-449147	16/392	1.01E-18	0.000	43.87234043	1817.879507	SYK;SRC;BRAF;PIK3R1;TYK2;EGFR;HCK;ERBB3;ERBB4;ERBB2;JAK2;JAK3;FGFR3;FGFR2;JAK1;FGFR1
Fc epsilon receptor (FCERI) signaling Homo sapiens R-HSA-2454202	16/395	1.14E-18	0.000	43.51840022	1797.91999	SYK;SRC;TXK;INSR;BRAF;PIK3R1;EGFR;ERBB3;ERBB4;ERBB2;JAK2;JAK3;FGFR3;FGFR2;JAK1;FGFR1
Interleukin-2 signaling Homo sapiens R-HSA-451927	14/252	3.24E-18	0.000	55.25770308	2225.264603	SYK;SRC;BRAF;PIK3R1;EGFR;ERBB3;ERBB4;ERBB2;JAK2;JAK3;FGFR3;FGFR2;JAK1;FGFR1
DAP12 signaling Homo sapiens R-HSA-2424491	15/344	5.98E-18	0.000	44.76291793	1775.187564	SYK;SRC;INSR;BRAF;PIK3R1;EGFR;ERBB3;ERBB4;ERBB2;JAK2;JAK3;FGFR3;FGFR2;JAK1;FGFR1
DAP12 interactions Homo sapiens R-HSA-2172127	15/359	1.13E-17	0.000	42.77834302	1669.146141	SYK;SRC;INSR;BRAF;PIK3R1;EGFR;ERBB3;ERBB4;ERBB2;JAK2;JAK3;FGFR3;FGFR2;JAK1;FGFR1
Insulin receptor signalling cascade Homo sapiens R-HSA-74751	14/287	2.02E-17	0.000	48.08791209	1848.451211	SRC;INSR;BRAF;PIK3R1;EGFR;ERBB3;ERBB4;ERBB2;JAK2;JAK3;FGFR3;FGFR2;JAK1;FGFR1
Signaling by Insulin receptor Homo sapiens R-HSA-74752	14/311	6.24E-17	0.000	44.14814815	1647.309223	SRC;INSR;BRAF;PIK3R1;EGFR;ERBB3;ERBB4;ERBB2;JAK2;JAK3;FGFR3;FGFR2;JAK1;FGFR1
VEGFA-VEGFR2 Pathway Homo sapiens R-HSA-4420097	14/320	9.30E-17	0.000	42.83006536	1581.040924	ROCK2;SRC;BRAF;PIK3R1;EGFR;ERBB3;ERBB4;ERBB2;JAK2;JAK3;FGFR3;FGFR2;JAK1;FGFR1
MAPK1/MAPK3 signaling Homo sapiens R-HSA-5684996	13/241	9.52E-17	0.000	51.15251196	1887.044521	SRC;BRAF;TYK2;EGFR;ERBB3;ERBB4;ERBB2;JAK2;JAK3;FGFR3;FGFR2;JAK1;FGFR1

Interleukin receptor SHC signaling Homo sapiens R-HSA-912526	13/245	1.18E-16	0.000	50.26038401	1843.309999	SRC;BRAF;PIK3R1;EGFR;ERBB3;ERBB4;ERBB2;JAK2;JAK3;FGFR3;FGFR2;JAK1;FGFR1
Signaling by VEGF Homo sapiens R-HSA-194138	14/328	1.31E-16	0.000	41.72186837	1525.743187	ROCK2;SRC;BRAF;PIK3R1;EGFR;ERBB3;ERBB4;ERBB2;JAK2;JAK3;FGFR3;FGFR2;JAK1;FGFR1
Downstream signaling of activated FGFR2 Homo sapiens R-HSA-5654696	14/329	1.37E-16	0.000	41.58730159	1519.055027	SRC;INSR;BRAF;PIK3R1;EGFR;ERBB3;ERBB4;ERBB2;JAK2;JAK3;FGFR3;FGFR2;JAK1;FGFR1
Downstream signaling of activated FGFR4 Homo sapiens R-HSA-5654716	14/329	1.37E-16	0.000	41.58730159	1519.055027	SRC;INSR;BRAF;PIK3R1;EGFR;ERBB3;ERBB4;ERBB2;JAK2;JAK3;FGFR3;FGFR2;JAK1;FGFR1
Downstream signaling of activated FGFR3 Homo sapiens R-HSA-5654708	14/329	1.37E-16	0.000	41.58730159	1519.055027	SRC;INSR;BRAF;PIK3R1;EGFR;ERBB3;ERBB4;ERBB2;JAK2;JAK3;FGFR3;FGFR2;JAK1;FGFR1
Signaling by ERBB4 Homo sapiens R-HSA-1236394	14/330	1.43E-16	0.000	41.4535865	1512.414961	SRC;INSR;BRAF;PIK3R1;EGFR;ERBB3;ERBB4;ERBB2;JAK2;JAK3;FGFR3;FGFR2;JAK1;FGFR1
PI5P. PP2A and IER3 Regulate PI3K/AKT Signaling Homo sapiens R-HSA-6811558	10/83	1.46E-16	0.000	108.9972603	3974.472929	ERBB3;SRC;ERBB4;INSR;ERBB2;PIK3R1;FGFR3;EGFR;FGFR2;FGFR1
Signaling by FGFR4 Homo sapiens R-HSA-5654743	14/332	1.55E-16	0.000	41.18867925	1499.277157	SRC;INSR;BRAF;PIK3R1;EGFR;ERBB3;ERBB4;ERBB2;JAK2;JAK3;FGFR3;FGFR2;JAK1;FGFR1
Downstream signaling of activated FGFR1 Homo sapiens R-HSA-5654687	14/332	1.55E-16	0.000	41.18867925	1499.277157	SRC;INSR;BRAF;PIK3R1;EGFR;ERBB3;ERBB4;ERBB2;JAK2;JAK3;FGFR3;FGFR2;JAK1;FGFR1
Signaling by FGFR3 Homo sapiens R-HSA-5654741	14/333	1.62E-16	0.000	41.05747126	1492.778457	SRC;INSR;BRAF;PIK3R1;EGFR;ERBB3;ERBB4;ERBB2;JAK2;JAK3;FGFR3;FGFR2;JAK1;FGFR1
Signaling by FGFR1 Homo sapiens R-HSA-5654736	14/336	1.84E-16	0.000	40.66873706	1473.557531	SRC;INSR;BRAF;PIK3R1;EGFR;ERBB3;ERBB4;ERBB2;JAK2;JAK3;FGFR3;FGFR2;JAK1;FGFR1
Downstream signal transduction Homo sapiens R-HSA-186763	14/341	2.26E-16	0.000	40.03669725	1442.41271	SRC;INSR;BRAF;PIK3R1;EGFR;ERBB3;ERBB4;ERBB2;JAK2;JAK3;FGFR3;FGFR2;JAK1;FGFR1
Negative regulation of the PI3K/AKT network Homo sapiens R-HSA-199418	10/90	3.40E-16	0.000	99.425	3541.189994	ERBB3;SRC;ERBB4;INSR;ERBB2;PIK3R1;FGFR3;EGFR;FGFR2;FGFR1
Signaling by EGFR Homo sapiens R-HSA-177929	14/355	3.95E-16	0.000	38.3655914	1360.715523	SRC;INSR;BRAF;PIK3R1;EGFR;ERBB3;ERBB4;ERBB2;JAK2;JAK3;FGFR3;FGFR2;JAK1;FGFR1
Signaling by FGFR2 Homo sapiens R-HSA-5654738	14/361	4.99E-16	0.000	37.69068204	1327.994762	SRC;INSR;BRAF;PIK3R1;EGFR;ERBB3;ERBB4;ERBB2;JAK2;JAK3;FGFR3;FGFR2;JAK1;FGFR1
Signaling by PDGF Homo sapiens R-HSA-186797	14/364	5.60E-16	0.000	37.36190476	1312.113634	SRC;INSR;BRAF;PIK3R1;EGFR;ERBB3;ERBB4;ERBB2;JAK2;JAK3;FGFR3;FGFR2;JAK1;FGFR1
Signaling by FGFR Homo sapiens R-HSA-190236	14/366	6.04E-16	0.000	37.14583333	1301.697731	SRC;INSR;BRAF;PIK3R1;EGFR;ERBB3;ERBB4;ERBB2;JAK2;JAK3;FGFR3;FGFR2;JAK1;FGFR1
MAPK family signaling cascades Homo sapiens R-HSA-5683057	13/284	8.10E-16	0.000	42.94230124	1492.244021	SRC;BRAF;TYK2;EGFR;ERBB3;ERBB4;ERBB2;JAK2;JAK3;FGFR3;FGFR2;JAK1;FGFR1
IRS-mediated signalling Homo sapiens R-HSA-112399	13/284	8.10E-16	0.000	42.94230124	1492.244021	SRC;BRAF;PIK3R1;EGFR;ERBB3;ERBB4;ERBB2;JAK2;JAK3;FGFR3;FGFR2;JAK1;FGFR1
NGF signalling via TRKA from the plasma membrane Homo sapiens R-HSA-187037	14/374	8.16E-16	0.000	36.30555556	1261.352801	SRC;INSR;BRAF;PIK3R1;EGFR;ERBB3;ERBB4;ERBB2;JAK2;JAK3;FGFR3;FGFR2;JAK1;FGFR1
IGF1R signaling cascade Homo sapiens R-HSA-2428924	13/288	9.71E-16	0.000	42.30909091	1462.555817	SRC;BRAF;PIK3R1;EGFR;ERBB3;ERBB4;ERBB2;JAK2;JAK3;FGFR3;FGFR2;JAK1;FGFR1
Signaling by Type 1 Insulin-like Growth Factor 1 Receptor (IGF1R) Homo sapiens R-HSA-2404192	13/288	9.71E-16	0.000	42.30909091	1462.555817	SRC;BRAF;PIK3R1;EGFR;ERBB3;ERBB4;ERBB2;JAK2;JAK3;FGFR3;FGFR2;JAK1;FGFR1
IRS-related events triggered by IGF1R Homo sapiens R-HSA-2428928	13/288	9.71E-16	0.000	42.30909091	1462.555817	SRC;BRAF;PIK3R1;EGFR;ERBB3;ERBB4;ERBB2;JAK2;JAK3;FGFR3;FGFR2;JAK1;FGFR1
FCERI mediated MAPK activation Homo sapiens R-HSA-2871796	13/289	1.02E-15	0.000	42.15365613	1455.286076	SYK;SRC;BRAF;EGFR;ERBB3;ERBB4;ERBB2;JAK2;JAK3;FGFR3;FGFR2;JAK1;FGFR1
Cytokine Signaling in Immune system Homo sapiens R-HSA-1280215	16/620	1.41E-15	0.000	26.99337748	923.0016881	SYK;SRC;BRAF;PIK3R1;TYK2;EGFR;HCK;ERBB3;ERBB4;ERBB2;JAK2;JAK3;FGFR3;FGFR2;JAK1;FGFR1
Role of LAT2/NTAL/LAB on calcium mobilization Homo sapiens R-HSA-2730905	11/162	2.46E-15	0.000	60.14183223	2023.009361	ERBB3;SYK;SRC;ERBB4;INSR;ERBB2;PIK3R1;FGFR3;EGFR;FGFR2;FGFR1
GRB2 events in EGFR signaling Homo sapiens R-HSA-179812	12/235	3.43E-15	0.000	46.18912069	1538.345703	ERBB3;SRC;ERBB4;ERBB2;BRAF;JAK2;JAK3;FGFR3;EGFR;FGFR2;JAK1;FGFR1
SHC1 events in EGFR signaling Homo sapiens R-HSA-180336	12/235	3.43E-15	0.000	46.18912069	1538.345703	ERBB3;SRC;ERBB4;ERBB2;BRAF;JAK2;JAK3;FGFR3;EGFR;FGFR2;JAK1;FGFR1
SOS-mediated signalling Homo sapiens R-HSA-112412	12/235	3.43E-15	0.000	46.18912069	1538.345703	ERBB3;SRC;ERBB4;ERBB2;BRAF;JAK2;JAK3;FGFR3;EGFR;FGFR2;JAK1;FGFR1
SHC1 events in ERBB4 signaling Homo sapiens R-HSA-1250347	12/235	3.43E-15	0.000	46.18912069	1538.345703	ERBB3;SRC;ERBB4;ERBB2;BRAF;JAK2;JAK3;FGFR3;EGFR;FGFR2;JAK1;FGFR1
RAF/MAP kinase cascade Homo sapiens R-HSA-5673001	12/235	3.43E-15	0.000	46.18912069	1538.345703	ERBB3;SRC;ERBB4;ERBB2;BRAF;JAK2;JAK3;FGFR3;EGFR;FGFR2;JAK1;FGFR1
FRS-mediated FGFR2 signaling Homo sapiens R-HSA-5654700	12/236	3.61E-15	0.000	45.98059006	1529.050213	ERBB3;SRC;ERBB4;ERBB2;BRAF;JAK2;JAK3;FGFR3;EGFR;FGFR2;JAK1;FGFR1
FRS-mediated FGFR4 signaling Homo sapiens R-HSA-5654712	12/236	3.61E-15	0.000	45.98059006	1529.050213	ERBB3;SRC;ERBB4;ERBB2;BRAF;JAK2;JAK3;FGFR3;EGFR;FGFR2;JAK1;FGFR1
FRS-mediated FGFR3 signaling Homo sapiens R-HSA-5654706	12/236	3.61E-15	0.000	45.98059006	1529.050213	ERBB3;SRC;ERBB4;ERBB2;BRAF;JAK2;JAK3;FGFR3;EGFR;FGFR2;JAK1;FGFR1
FRS-mediated FGFR1 signaling Homo sapiens R-HSA-5654693	12/236	3.61E-15	0.000	45.98059006	1529.050213	ERBB3;SRC;ERBB4;ERBB2;BRAF;JAK2;JAK3;FGFR3;EGFR;FGFR2;JAK1;FGFR1
Innate Immune System Homo sapiens R-HSA-168249	17/807	3.87E-15	0.000	22.92369902	760.7237858	SYK;SRC;TXK;INSR;BRAF;PIK3R1;EGFR;HCK;ERBB3;ERBB4;ERBB2;JAK2;JAK3;FGFR3;FGFR2;JAK1;FGFR1
ARMS-mediated activation Homo sapiens R-HSA-170984	12/239	4.21E-15	0.000	45.36602183	1501.717726	ERBB3;SRC;ERBB4;ERBB2;BRAF;JAK2;JAK3;FGFR3;EGFR;FGFR2;JAK1;FGFR1
Signalling to p38 via RIT and RIN Homo sapiens R-HSA-187706	12/239	4.21E-15	0.000	45.36602183	1501.717726	ERBB3;SRC;ERBB4;ERBB2;BRAF;JAK2;JAK3;FGFR3;EGFR;FGFR2;JAK1;FGFR1

Frs2-mediated activation Homo sapiens R-HSA-170968	12/240	4.42E-15	0.000	45.16475973	1492.787213	ERBB3;SRC;ERBB4;ERBB2;BRAF;JAK2;JAK3;FGFR3;EGFR;FGFR2;JAK1;FGFR1
Prolonged ERK activation events Homo sapiens R-HSA-169893	12/242	4.89E-15	0.000	44.76748582	1475.189092	ERBB3;SRC;ERBB4;ERBB2;BRAF;JAK2;JAK3;FGFR3;EGFR;FGFR2;JAK1;FGFR1
Signaling by Leptin Homo sapiens R-HSA-2586552	12/243	5.14E-15	0.000	44.57142857	1466.519041	ERBB3;SRC;ERBB4;ERBB2;BRAF;JAK2;JAK3;FGFR3;EGFR;FGFR2;JAK1;FGFR1
Signalling to RAS Homo sapiens R-HSA-167044	12/246	5.95E-15	0.000	43.99331104	1441.010724	ERBB3;SRC;ERBB4;ERBB2;BRAF;JAK2;JAK3;FGFR3;EGFR;FGFR2;JAK1;FGFR1
VEGFR2 mediated cell proliferation Homo sapiens R-HSA-5218921	12/248	6.56E-15	0.000	43.61606485	1424.411965	ERBB3;SRC;ERBB4;ERBB2;BRAF;JAK2;JAK3;FGFR3;EGFR;FGFR2;JAK1;FGFR1
PI-3K cascade:FGFR1 Homo sapiens R-HSA-5654689	10/122	7.91E-15	0.000	70.90357143	2302.327214	ERBB3;SRC;ERBB4;INSR;ERBB2;PIK3R1;FGFR3;EGFR;FGFR2;FGFR1
PI-3K cascade:FGFR3 Homo sapiens R-HSA-5654710	10/122	7.91E-15	0.000	70.90357143	2302.327214	ERBB3;SRC;ERBB4;INSR;ERBB2;PIK3R1;FGFR3;EGFR;FGFR2;FGFR1
PI3K events in ERBB4 signaling Homo sapiens R-HSA-1250342	10/122	7.91E-15	0.000	70.90357143	2302.327214	ERBB3;SRC;ERBB4;INSR;ERBB2;PIK3R1;FGFR3;EGFR;FGFR2;FGFR1
PIP3 activates AKT signaling Homo sapiens R-HSA-1257604	10/122	7.91E-15	0.000	70.90357143	2302.327214	ERBB3;SRC;ERBB4;INSR;ERBB2;PIK3R1;FGFR3;EGFR;FGFR2;FGFR1
PI-3K cascade:FGFR4 Homo sapiens R-HSA-5654720	10/122	7.91E-15	0.000	70.90357143	2302.327214	ERBB3;SRC;ERBB4;INSR;ERBB2;PIK3R1;FGFR3;EGFR;FGFR2;FGFR1
PI-3K cascade:FGFR2 Homo sapiens R-HSA-5654695	10/122	7.91E-15	0.000	70.90357143	2302.327214	ERBB3;SRC;ERBB4;INSR;ERBB2;PIK3R1;FGFR3;EGFR;FGFR2;FGFR1
Signalling to ERKs Homo sapiens R-HSA-187687	12/253	8.33E-15	0.000	42.70034277	1384.275438	ERBB3;SRC;ERBB4;ERBB2;BRAF;JAK2;JAK3;FGFR3;EGFR;FGFR2;JAK1;FGFR1
GAB1 signalosome Homo sapiens R-HSA-180292	10/125	1.01E-14	0.000	69.04347826	2224.746748	ERBB3;SRC;ERBB4;INSR;ERBB2;PIK3R1;FGFR3;EGFR;FGFR2;FGFR1
PI3K/AKT activation Homo sapiens R-HSA-198203	10/125	1.01E-14	0.000	69.04347826	2224.746748	ERBB3;SRC;ERBB4;INSR;ERBB2;PIK3R1;FGFR3;EGFR;FGFR2;FGFR1
Signalling by NGF Homo sapiens R-HSA-166520	14/450	1.05E-14	0.000	29.86085627	961.1180073	SRC;INSR;BRAF;PIK3R1;EGFR;ERBB3;ERBB4;ERBB2;JAK2;JAK3;FGFR3;FGFR2;JAK1;FGFR1
Signal Transduction Homo sapiens R-HSA-162582	24/2465	1.45E-14	0.000	15.66332725	499.1302383	SYK;ROCK2;SRC;LRRK2;INSR;PLK1;BRAF;PTK6;PIK3R1;TYK2;EGFR;CDK8;FER;ERBB3;FNTA;ERBB4;FES;ERBB2;JAK2;JAK3;FGFR3;FGFR2;JAK1;FGFR1
NCAM signaling for neurite out-growth Homo sapiens R-HSA-375165	12/266	1.52E-14	0.000	40.48818898	1288.248436	ERBB3;SRC;ERBB4;ERBB2;BRAF;JAK2;JAK3;FGFR3;EGFR;FGFR2;JAK1;FGFR1
Developmental Biology Homo sapiens R-HSA-1266738	16/786	5.62E-14	0.000	20.9924812	640.4693395	CDK19;ROCK2;SRC;BRAF;EGFR;CDK8;ERBB3;ERBB4;FES;ERBB2;JAK2;JAK3;FGFR3;FGFR2;JAK1;FGFR1
Axon guidance Homo sapiens R-HSA-422475	14/515	6.68E-14	0.000	25.9001996	785.7260742	ROCK2;SRC;BRAF;EGFR;ERBB3;ERBB4;FES;ERBB2;JAK2;JAK3;FGFR3;FGFR2;JAK1;FGFR1
Constitutive Signaling by Aberrant PI3K in Cancer Homo sapiens R-HSA-2219530	8/61	1.03E-13	0.000	111.3179595	3329.374156	ERBB3;ERBB4;ERBB2;PIK3R1;FGFR3;EGFR;FGFR2;FGFR1
Signaling by the B Cell Receptor (BCR) Homo sapiens R-HSA-983705	11/233	1.38E-13	0.000	40.7606982	1206.951297	ERBB3;SYK;SRC;ERBB4;INSR;ERBB2;PIK3R1;FGFR3;EGFR;FGFR2;FGFR1
Gastrin-CREB signalling pathway via PKC and MAPK Homo sapiens R-HSA-881907	13/432	1.79E-13	0.000	27.56541549	809.1560715	SRC;BRAF;PIK3R1;EGFR;ERBB3;ERBB4;ERBB2;JAK2;JAK3;FGFR3;FGFR2;JAK1;FGFR1
Downstream signaling events of B Cell Receptor (BCR) Homo sapiens R-HSA-1168372	10/192	7.83E-13	0.000	43.47912088	1212.021687	ERBB3;SRC;ERBB4;INSR;ERBB2;PIK3R1;FGFR3;EGFR;FGFR2;FGFR1
Immune System Homo sapiens R-HSA-168256	19/1547	8.34E-13	0.000	14.32849313	398.5120803	SYK;SRC;TXK;INSR;BRAF;PIK3R1;TYK2;EGFR;HCK;ZAP70;ERBB3;ERBB4;ERBB2;JAK2;JAK3;FGFR3;FGFR2;JAK1;FGFR1
Signaling by ERBB2 Homo sapiens R-HSA-1227986	7/45	1.15E-12	0.000	131.0986842	3604.339983	ERBB3;SRC;ERBB4;ERBB2;PTK6;PIK3R1;EGFR
PI3K/AKT Signaling in Cancer Homo sapiens R-HSA-2219528	8/86	1.79E-12	0.000	75.54415954	2043.267031	ERBB3;ERBB4;ERBB2;PIK3R1;FGFR3;EGFR;FGFR2;FGFR1
ERBB2 Activates PTK6 Signaling Homo sapiens R-HSA-8847993	5/13	1.55E-11	0.000	415.7708333	10348.21758	ERBB3;ERBB4;ERBB2;PTK6;EGFR
PI3K events in ERBB2 signaling Homo sapiens R-HSA-1963642	5/16	5.25E-11	0.000	302.3333333	7156.522699	ERBB3;ERBB4;ERBB2;PIK3R1;EGFR
Diseases of signal transduction Homo sapiens R-HSA-5663202	9/288	1.20E-09	0.000	24.42431762	501.7986476	CDK8;ERBB3;ERBB4;ERBB2;PIK3R1;FGFR3;EGFR;FGFR2;FGFR1
Signaling by PTK6 Homo sapiens R-HSA-8848021	6/67	1.69E-09	0.000	67.5093273	1363.588729	ERBB3;ERBB4;LRRK2;ERBB2;PTK6;EGFR
Adaptive Immune System Homo sapiens R-HSA-1280218	12/762	3.20E-09	0.000	13.36695652	261.4753039	ZAP70;ERBB3;SYK;SRC;ERBB4;INSR;ERBB2;PIK3R1;FGFR3;EGFR;FGFR2;FGFR1
ERBB2 Regulates Cell Motility Homo sapiens R-HSA-6785631	4/15	1.06E-08	0.000	234.0645161	4298.501262	ERBB3;ERBB4;ERBB2;EGFR
Signaling by GPCR Homo sapiens R-HSA-372790	14/1293	1.33E-08	0.000	9.739900964	176.6247481	ROCK2;SRC;BRAF;PIK3R1;EGFR;ERBB3;ERBB4;ERBB2;JAK2;JAK3;FGFR3;FGFR2;JAK1;FGFR1
SHC1 events in ERBB2 signaling Homo sapiens R-HSA-1250196	4/18	2.36E-08	0.000	183.8801843	3229.128859	ERBB3;ERBB4;ERBB2;EGFR
GPVI-mediated activation cascade Homo sapiens R-HSA-114604	5/53	3.29E-08	0.000	69.15625	1191.526964	SYK;PIK3R1;JAK2;JAK3;JAK1
PTK6 promotes HIF1A stabilization Homo sapiens R-HSA-8857538	3/7	1.71E-07	0.000	467.8359375	7289.664133	LRRK2;PTK6;EGFR
Disease Homo sapiens R-HSA-1643685	10/725	2.97E-07	0.000	10.76923077	161.8523808	CDK8;HCK;ERBB3;ERBB4;ERBB2;PIK3R1;FGFR3;EGFR;FGFR2;FGFR1
MAPK1 (ERK2) activation Homo sapiens R-HSA-112411	3/9	4.09E-07	0.000	311.859375	4587.011006	TYK2;JAK2;JAK1
MAPK3 (ERK1) activation Homo sapiens R-HSA-110056	3/10	5.84E-07	0.000	267.2946429	3836.509036	TYK2;JAK2;JAK1
Interleukin-6 signaling Homo sapiens R-HSA-1059683	3/11	8.02E-07	0.000	233.8710938	3282.579863	TYK2;JAK2;JAK1
Interleukin-7 signaling Homo sapiens R-HSA-1266695	3/11	8.02E-07	0.000	233.8710938	3282.579863	PIK3R1;JAK3;JAK1
G beta:gamma signalling through PI3Kgamma Homo sapiens R-HSA-	4/48	1.45E-06	0.000	58.41935484	785.493754	PIK3R1;JAK2;JAK3;JAK1

392451						
G-protein beta:gamma signalling Homo sapiens R-HSA-397795	4/51	1.85E-06	0.000	54.68222375	721.7659866	PIK3R1;JAK2;JAK3;JAK1
GRB2 events in ERBB2 signaling Homo sapiens R-HSA-1963640	3/16	2.71E-06	0.000	143.8846154	1844.578682	ERBB4;ERBB2;EGFR
Regulation of signaling by CBL Homo sapiens R-HSA-912631	3/18	3.93E-06	0.000	124.6875	1551.831318	HCK;SYK;PIK3R1
Signaling by FGFR in disease Homo sapiens R-HSA-1226099	4/63	4.35E-06	0.000	43.53417168	537.4526891	PIK3R1;FGFR3;FGFR2;FGFR1
Platelet activation. signaling and aggregation Homo sapiens R-HSA-76002	6/253	4.61E-06	0.000	16.51654335	202.9497903	SYK;SRC;PIK3R1;JAK2;JAK3;JAK1
IL-6-type cytokine receptor ligand interactions Homo sapiens R-HSA-6788467	3/20	5.48E-06	0.000	110.0073529	1332.606216	TYK2;JAK2;JAK1
RAF-independent MAPK1/3 activation Homo sapiens R-HSA-112409	3/23	8.49E-06	0.000	93.4921875	1091.696242	TYK2;JAK2;JAK1
PI3K Cascade Homo sapiens R-HSA-109704	4/79	1.08E-05	0.000	34.21935484	391.4748995	PIK3R1;FGFR3;FGFR2;FGFR1
RAF activation Homo sapiens R-HSA-5673000	3/25	1.10E-05	0.000	84.984375	970.3433957	SRC;BRAF;JAK2
Interleukin-6 family signaling Homo sapiens R-HSA-6783589	3/27	1.40E-05	0.000	77.89453125	870.8542586	TYK2;JAK2;JAK1
Negative regulation of FGFR3 signaling Homo sapiens R-HSA-5654732	3/29	1.74E-05	0.000	71.89543269	787.9584422	SRC;BRAF;FGFR3
Negative regulation of FGFR1 signaling Homo sapiens R-HSA-5654726	3/32	2.35E-05	0.000	64.44827586	686.876629	SRC;BRAF;FGFR1
Negative regulation of FGFR2 signaling Homo sapiens R-HSA-5654727	3/34	2.83E-05	0.000	60.28427419	631.3276883	SRC;BRAF;FGFR2
GRB7 events in ERBB2 signaling Homo sapiens R-HSA-1306955	2/5	2.97E-05	0.000	403.2727273	4204.515497	ERBB3;ERBB2
Fcgamma receptor (FCGR) dependent phagocytosis Homo sapiens R-HSA-2029480	4/120	5.59E-05	0.000	22.07897664	216.2016098	HCK;SYK;SRC;PIK3R1
FCGR activation Homo sapiens R-HSA-2029481	3/49	8.56E-05	0.000	40.59578804	380.2169353	HCK;SYK;SRC
GP1b-IX-V activation signalling Homo sapiens R-HSA-430116	2/10	1.33E-04	0.000	151.1893939	1349.727435	SRC;PIK3R1
Signaling by FGFR3 fusions in cancer Homo sapiens R-HSA-8853334	2/10	1.33E-04	0.000	151.1893939	1349.727435	PIK3R1;FGFR3
Semaphorin interactions Homo sapiens R-HSA-373755	3/67	2.18E-04	0.001	29.15185547	245.8059719	ROCK2;FES;ERBB2
Downregulation of ERBB2:ERBB3 signaling Homo sapiens R-HSA-1358803	2/13	2.29E-04	0.001	109.9393939	921.3628505	ERBB3;ERBB2
Regulation of IFNG signaling Homo sapiens R-HSA-877312	2/14	2.67E-04	0.001	100.7727273	829.1167815	JAK2;JAK1
Signaling by EGFRvIII in Cancer Homo sapiens R-HSA-5637812	2/15	3.08E-04	0.001	93.01631702	752.0916572	PIK3R1;EGFR
Constitutive Signaling by EGFRvIII Homo sapiens R-HSA-5637810	2/15	3.08E-04	0.001	93.01631702	752.0916572	PIK3R1;EGFR
Spry regulation of FGF signaling Homo sapiens R-HSA-1295596	2/16	3.52E-04	0.001	86.36796537	686.8978438	SRC;BRAF
Hemostasis Homo sapiens R-HSA-109582	6/552	3.53E-04	0.001	7.358469117	58.48901632	SYK;SRC;PIK3R1;JAK2;JAK3;JAK1
Constitutive Signaling by Ligand-Responsive EGFR Cancer Variants Homo sapiens R-HSA-1236382	2/19	4.99E-04	0.001	71.11586453	540.6425814	PIK3R1;EGFR
Signaling by Ligand-Responsive EGFR Variants in Cancer Homo sapiens R-HSA-5637815	2/19	4.99E-04	0.001	71.11586453	540.6425814	PIK3R1;EGFR
Signaling by EGFR in Cancer Homo sapiens R-HSA-1643713	2/19	4.99E-04	0.001	71.11586453	540.6425814	PIK3R1;EGFR
Signal transduction by L1 Homo sapiens R-HSA-445144	2/21	6.12E-04	0.002	63.62360447	470.7529763	EGFR;FGFR1
L1CAM interactions Homo sapiens R-HSA-373760	3/96	6.27E-04	0.002	20.03225806	147.7145778	SRC;EGFR;FGFR1
Signaling by FGFR3 point mutants in cancer Homo sapiens R-HSA-8853338	2/22	6.72E-04	0.002	60.43939394	441.498777	PIK3R1;FGFR3
Signaling by FGFR3 in disease Homo sapiens R-HSA-5655332	2/22	6.72E-04	0.002	60.43939394	441.498777	PIK3R1;FGFR3
Sema4D induced cell migration and growth-cone collapse Homo sapiens R-HSA-416572	2/24	8.01E-04	0.002	54.93939394	391.6646216	ROCK2;ERBB2
Regulation of IFNA signaling Homo sapiens R-HSA-912694	2/25	8.70E-04	0.002	52.54808959	370.2930792	TYK2;JAK1
Integrin alphaIIb beta3 signaling Homo sapiens R-HSA-354192	2/27	0.001015943	0.003	48.33939394	333.1521161	SYK;SRC
Sema4D in semaphorin signaling Homo sapiens R-HSA-400685	2/27	0.001015943	0.003	48.33939394	333.1521161	ROCK2;ERBB2
Negative regulation of FGFR4 signaling Homo sapiens R-HSA-	2/31	0.001340012	0.003	41.66353187	275.6074457	SRC;BRAF

5654733						
FGFR1 mutant receptor activation Homo sapiens R-HSA-1839124	2/31	0.001340012	0.003	41.66353187	275.6074457	PIK3R1;FGFR1
CD28 co-stimulation Homo sapiens R-HSA-389356	2/33	0.001518228	0.004	38.971652	252.9342688	SRC;PIK3R1
EPHA-mediated growth cone collapse Homo sapiens R-HSA-3928663	2/34	0.001611348	0.004	37.75189394	242.7704971	SRC;ROCK2
Platelet Aggregation (Plug Formation) Homo sapiens R-HSA-76009	2/37	0.001906654	0.005	34.51082251	216.1207577	SYK;SRC
MAP2K and MAPK activation Homo sapiens R-HSA-5674135	2/38	0.002010374	0.005	33.55050505	208.3296666	SRC;BRAF
Signaling by FGFR1 in disease Homo sapiens R-HSA-5655302	2/38	0.002010374	0.005	33.55050505	208.3296666	PIK3R1;FGFR1
Nuclear signaling by ERBB4 Homo sapiens R-HSA-1251985	2/39	0.002116721	0.005	32.64209664	201.0063514	ERBB4;JAK2
EPHB-mediated forward signaling Homo sapiens R-HSA-3928662	2/42	0.002451434	0.006	30.18939394	181.4709231	ROCK2;SRC
Signaling by FGFR2 in disease Homo sapiens R-HSA-5655253	2/43	0.0025682	0.006	29.45158906	175.6654747	PIK3R1;FGFR2
Antigen activates B Cell Receptor (BCR) leading to generation of second messengers Homo sapiens R-HSA-983695	2/47	0.003061	0.007	26.82828283	155.3092959	SYK;PIK3R1
Interferon Signaling Homo sapiens R-HSA-913531	3/196	0.00481639	0.011	9.604274611	51.24582285	TYK2;JAK2;JAK1
Role of phospholipids in phagocytosis Homo sapiens R-HSA-2029485	2/60	0.004941169	0.012	20.8014629	110.4589595	SYK;PIK3R1
FCERI mediated Ca+2 mobilization Homo sapiens R-HSA-2871809	2/62	0.005267417	0.012	20.10606061	105.4807208	SYK;TXK
Interferon alpha/beta signaling Homo sapiens R-HSA-909733	2/68	0.006303914	0.015	18.27272727	92.58031757	TYK2;JAK1
Costimulation by the CD28 family Homo sapiens R-HSA-388841	2/72	0.007042367	0.016	17.22510823	85.36437955	SRC;PIK3R1
G alpha (12/13) signalling events Homo sapiens R-HSA-416482	2/75	0.007620742	0.017	16.51473641	80.54041382	ROCK2;PIK3R1
Transcriptional regulation of white adipocyte differentiation Homo sapiens R-HSA-381340	2/79	0.008424198	0.019	15.65367965	74.7721024	CDK8;CDK19
PTK6 Regulates Proteins Involved in RNA Processing Homo sapiens R-HSA-8849468	1/5	0.008720203	0.019	146.7720588	696.0096582	PTK6
IRS activation Homo sapiens R-HSA-74713	1/5	0.008720203	0.019	146.7720588	696.0096582	INSR
PTK6 Expression Homo sapiens R-HSA-8849473	1/5	0.008720203	0.019	146.7720588	696.0096582	PTK6
RHO GTPase Effectors Homo sapiens R-HSA-195258	3/255	0.009923264	0.022	7.333705357	33.82945386	ROCK2;SRC;PLK1
