# Supplementary material for: Relationship of micro-RNA, mRNA and eIF Expression in Tamoxifen-Adapted MCF-7 Breast Cancer Cells: Impact of miR-1972 on Gene Expression, Proliferation and Migration
Source: Biomolecules. 2022 Jun 29;12(7):916. doi: 10.3390/biom12070916 (PMC9312698; doi:10.3390/biom12070916)
Supplement: Supplementary file 1 [file biomolecules-12-00916-s001.zip › Table S4 nCounter Results.pdf]

pam50		normalized counts									
	gene	MCF-7	TAM	logFc TAM/MCF-7	p	MCF-7 miR1972	logFc MCF-7 miR1972	p	TAM miR1972	logFc TAM miR1972	p
Luminal A	MAPT	546.19	508.80	-0.51	0.326	367.68	-0.57	0.003	384.46	-0.40	0.11
	NAT1	333.26	283.11	0.04	0.215	618.81	0.89	0.129	343.60	0.28	0.16
	SLC39A6	70892.83	6856.74	-3.44	0.001	66882.81	-0.08	0.489	6537.37	-0.07	0.21
	BIRC5	8339.69	10818.97	0.06	0.036	8337.49	0.00	0.985	8702.10	-0.31	0.06
	CDCA1	250.71	306.93	0.71	0.129	362.20	0.53	0.114	408.85	0.41	0.09
	CENPF	1141.37	1587.13	0.67	0.000	1381.64	0.28	0.169	1818.29	0.20	0.05
	EXO1	1221.95	1959.46	0.59	0.104	1414.14	0.21	0.080	1833.34	-0.10	0.49
	MYBL2	3018.42	2930.51	-0.09	0.406	3397.60	0.17	0.134	2829.99	-0.05	0.48
	PTTG1	8131.24	4014.87	-0.74	0.004	10681.48	0.39	0.122	4875.72	0.28	0.04
	UBE2C	8725.24	7139.99	-0.13	0.011	11872.11	0.44	0.021	7947.82	0.15	0.07
Luminal B	CXXC5	9914.69	4342.52	-1.11	0.006	8890.62	-0.16	0.305	4587.38	0.08	0.50
	KIF2C	1343.94	1580.62	0.19	0.078	1614.95	0.27	0.119	1533.14	-0.04	0.58
	MDM2	10010.39	12163.56	-0.66	0.030	5370.77	-0.90	0.005	6334.31	-0.94	0.02
	ORC6L	1601.75	2061.44	0.32	0.042	1885.28	0.24	0.114	2001.59	-0.04	0.31
	PGR	782.54	1.14	-9.44	0.009	828.12	0.08	0.718	1.13	-0.01	0.92
	BCL2	30.10	1.14	-2.41	0.046	14.51	-1.05	0.151	5.66	2.32	0.51
	CDH3	1245.00	1842.93	0.41	0.010	1405.59	0.18	0.155	1658.92	-0.15	0.14
	EGFR	1.35	165.91	7.44	0.046	24.47	4.18	0.035	234.22	0.50	0.13
	MKI67	3611.20	5684.00	0.58	0.031	3754.27	0.06	0.571	5390.96	-0.08	0.52
	PHGDH	2038.05	2893.81	0.67	0.016	2513.79	0.30	0.051	3240.15	0.16	0.08
Normal-like	KRT14	73.96	95.27	0.02	0.396	76.89	0.06	0.436	75.03	-0.34	0.41
	KRT17	1.35	1.14	-0.26	0.171	1.16	-0.22	0.332	1.13	-0.01	0.92

	KRT5	1.35	1.14	-0.26	0.171	1.16	-0.22	0.332	1.13	-0.01	0.92
	MLPH	2963.29	1141.84	-1.25	0.000	2479.03	-0.26	0.007	1249.29	0.13	0.05
	CCNB1	23152.43	20053.65	-0.35	0.088	23950.34	0.05	0.411	18212.90	-0.14	0.17
	CDC6	1773.17	2964.27	0.37	0.001	1918.39	0.11	0.385	2298.86	-0.37	0.00
	MMP11	40.73	58.92	0.89	0.188	68.80	0.76	0.127	75.35	0.35	0.22
	RRM2	16892.73	26042.69	0.97	0.011	25230.78	0.58	0.054	33203.74	0.35	0.00
	TYMS	4099.73	5803.24	1.44	0.080	8506.32	1.05	0.002	11144.89	0.94	0.03
	UBE2T	973.01	1224.22	0.68	0.055	1647.77	0.76	0.040	1562.72	0.35	0.08
HER2-enriched	BLVRA	3396.20	530.83	-2.25	0.005	4270.76	0.33	0.162	715.17	0.43	0.01
	ERBB2	785.79	1481.27	0.38	0.000	666.88	-0.24	0.144	1024.74	-0.53	0.06
	FGFR4	538.56	176.13	-0.81	0.003	669.72	0.31	0.017	307.47	0.80	0.13
	GRB7	426.96	684.45	0.43	0.065	426.31	0.00	0.993	574.81	-0.25	0.25
	BAG1	5533.10	6025.13	0.62	0.030	5765.71	0.06	0.328	8519.43	0.50	0.00
	CCNE1	1498.51	4865.70	1.68	0.008	1313.12	-0.19	0.270	4812.73	-0.02	0.83
	CDC20	3385.66	3761.93	-0.03	0.032	3743.88	0.15	0.147	3312.49	-0.18	0.02
	ACTR3B	1663.17	1543.53	0.00	0.084	1653.71	-0.01	0.763	1661.61	0.11	0.11
	MYC	6889.91	14714.49	0.79	0.010	4813.92	-0.52	0.010	11882.28	-0.31	0.03
	SFRP1	1.35	1.14	-0.26	0.171	1.16	-0.22	0.332	1.13	-0.01	0.92
Basal-like	ANLN	4598.01	5557.60	0.19	0.035	5877.85	0.35	0.025	5255.69	-0.08	0.07
	CEP55	2538.54	2681.69	0.36	0.327	3578.57	0.50	0.023	3263.35	0.28	0.02
	FOXC1	868.90	719.45	-0.18	0.024	1376.00	0.66	0.023	768.94	0.10	0.28
	KNTC2	2882.35	3231.61	0.39	0.145	4041.11	0.49	0.048	3765.64	0.22	0.10
	MELK	2221.79	2016.71	-0.19	0.106	2432.58	0.13	0.050	1942.40	-0.05	0.48
	MIA	1.35	1.14	-0.26	0.171	1.16	-0.22	0.332	1.13	-0.01	0.92
	ESR1	9725.30	3173.03	-1.71	0.009	7523.40	-0.37	0.136	2966.47	-0.10	0.44
	FOXA1	13826.26	11383.94	-0.85	0.021	10748.57	-0.36	0.015	7696.35	-0.56	0.00

GPR160	5971.36	7932.95	0.29	0.152	6025.83	0.01	0.876	7318.88	-0.12	0.48
TMEM45B	106.41	2366.38	4.03	0.015	225.66	1.08	0.180	1738.32	-0.44	0.04

Tam-gene set

Name	normalized counts<br>MCF-7	TAM	logFc TAM/MCF-7	p	MCF-7 miR1972	logFC MCF-7 / mir1972	p	TAM miR1972	logFc TAM miR1972	p
UBD	0.0273	0.0525	0.94	0.191	0.0052	-2.38	0.012	0.0057	-3.20	0.109
KLK11	0.0227	0.0007	-5.06	0.011	0.0418	0.88	0.213	0.0001	-3.48	0.157
LINC01213	0.0126	0.0838	2.73	0.006	0.0151	0.26	0.467	0.0671	-0.32	0.077
BMP5	0.0334	0.0003	-6.80	0.020	0.0659	0.98	0.026	0.0002	-0.40	0.685
VAV3	0.1432	0.0099	-3.86	0.001	0.1290	-0.15	0.068	0.0070	-0.51	0.342
PGK1	1.3372	1.6511	0.30	0.003	0.7797	-0.78	0.047	0.8317	-0.99	0.000
LINC00992	0.0004	0.0011	1.50	0.308	0.0005	0.35	0.843	0.0003	-1.73	0.129
SIM1	0.0018	0.0010	-0.86	0.307	0.0021	0.18	0.764	0.0005	-1.01	0.335
LOC101929547	0.0123	0.0009	-3.75	0.099	0.0262	1.09	0.032	0.0013	0.54	0.302
GPR68	0.1063	0.0019	-5.78	0.015	0.0941	-0.18	0.353	0.0021	0.14	0.511
KRT4	0.0023	0.0056	1.28	0.216	0.0164	2.84	0.190	0.0210	1.92	0.019
ZNF462	0.0063	0.0077	0.31	0.195	0.0030	-1.08	0.080	0.0047	-0.72	0.050
ARHGAP26	0.0180	0.1451	3.01	0.020	0.0111	-0.70	0.031	0.0612	-1.25	0.008
RPL13	1.0000	1.0000	0.00		1.0000	0.00		1.0000	0.00	
C1orf226	0.0427	0.0115	-1.89	0.022	0.0332	-0.36	0.160	0.0073	-0.66	0.109
DHRS3	0.3540	1.0164	1.52	0.003	0.4829	0.45	0.408	0.5867	-0.79	0.015
BPIFB1	0.0743	0.0427	-0.80	0.080	0.0446	-0.74	0.039	0.0166	-1.36	0.054
CLTC	2.2876	3.0055	0.39	0.024	1.6421	-0.48	0.125	1.9518	-0.62	0.050
LINC00632	0.0013	0.0016	0.34	0.436	0.0009	-0.45	0.051	0.0007	-1.14	0.158
CA2	0.6589	0.0047	-7.13	0.005	0.6125	-0.11	0.212	0.0026	-0.84	0.252
GUSB	0.2040	0.2419	0.25	0.007	0.2023	-0.01	0.808	0.1832	-0.40	0.015
FAXDC2	0.0311	0.0019	-4.00	0.002	0.0163	-0.94	0.101	0.0009	-1.13	0.403

BDKRB2	0.0342	0.0367	0.10	0.524	0.0160	-1.10	0.011	0.0086	-2.10	0.059
CEACAM6	0.0931	0.0930	0.00	0.999	0.1188	0.35	0.220	0.1070	0.20	0.400
GREB1	0.1148	0.0032	-5.16	0.012	0.0480	-1.26	0.011	0.0011	-1.52	0.092
EPAS1	0.2039	1.2428	2.61	0.005	0.0833	-1.29	0.041	0.3450	-1.85	0.002
GPER1	0.0699	0.0036	-4.29	0.035	0.0331	-1.08	0.022	0.0013	-1.42	0.108
DEFB1	0.0015	0.0022	0.57	0.281	0.0011	-0.38	0.411	0.0012	-0.92	0.185
FOLH1	0.0030	0.0032	0.07	0.767	0.0027	-0.17	0.515	0.0013	-1.26	0.111
AR	0.0438	0.0529	0.27	0.082	0.0405	-0.11	0.364	0.0382	-0.47	0.050
RAMP3	0.1101	0.0026	-5.42	0.038	0.1002	-0.14	0.709	0.0017	-0.57	0.262
RGS22	0.0026	0.0021	-0.33	0.514	0.0016	-0.72	0.315	0.0007	-1.52	0.013
GPX2	0.0348	0.0310	-0.17	0.519	0.0305	-0.19	0.685	0.0162	-0.93	0.071
ADAMTS9	0.0121	0.0043	-1.50	0.010	0.0046	-1.41	0.003	0.0019	-1.18	0.067
SPINK4	0.0144	0.0082	-0.81	0.046	0.0143	0.00	0.985	0.0040	-1.03	0.083
ACTB	23.4840	35.0097	0.58	0.011	18.7127	-0.33	0.251	20.5542	-0.77	0.004
PTGER4	0.0233	0.0112	-1.06	0.076	0.0172	-0.43	0.122	0.0072	-0.64	0.102
SOX3	0.0883	0.0015	-5.92	0.028	0.0828	-0.09	0.518	0.0015	0.04	0.833
TFF3	0.1555	0.0031	-5.66	0.020	0.0737	-1.08	0.012	0.0020	-0.63	0.224
BAMBI	1.7970	2.6266	0.55	0.042	1.4300	-0.33	0.062	1.4212	-0.89	0.002
TARP	0.0412	0.0026	-3.97	0.001	0.0820	0.99	0.010	0.0015	-0.81	0.446
SEMA5A	0.0174	0.3339	4.26	0.000	0.0120	-0.54	0.185	0.1800	-0.89	0.057
ABCA12	0.0237	0.0039	-2.58	0.017	0.0201	-0.24	0.246	0.0016	-1.35	0.161
AMIGO2	0.1437	0.7469	2.38	0.042	0.1654	0.20	0.021	0.4108	-0.86	0.028
XR_133213	0.0036	0.0034	-0.09	0.684	0.0033	-0.10	0.120	0.0027	-0.33	0.334
PIP	0.0025	0.0043	0.82	0.129	0.0028	0.21	0.171	0.0021	-1.04	0.065
LINC00683	0.0111	0.0038	-1.54	0.022	0.0144	0.38	0.232	0.0021	-0.86	0.238
HPRT1	0.4892	0.7879	0.69	0.076	0.5229	0.10	0.505	0.7089	-0.15	0.241
