# Supplementary material for: Relationship of micro-RNA, mRNA and eIF Expression in Tamoxifen-Adapted MCF-7 Breast Cancer Cells: Impact of miR-1972 on Gene Expression, Proliferation and Migration
Source: Biomolecules. 2022 Jun 29;12(7):916. doi: 10.3390/biom12070916 (PMC9312698; doi:10.3390/biom12070916)
Supplement: Supplementary file 1 [file biomolecules-12-00916-s001.zip › Table S5 eIF statistics.pdf]

Table S5 A. Relative expression of eIFs as determined by Western blotting. Log_2Fc relative to actin and MCF-7 is given.

eIF	MCF-7	Tam	MDA-MB-468	HS578T	SkBr3	MDA-MB-231	UACC3199	T47D
eIF2α	-0.03	-0.85	-0.51	-0.24	0.75	1.30	1.19	0.98
PeIF2 α	-0.04	0.82	-0.58	0.22	2.32	2.49	2.18	-0.39
peIF2α/eIF2α	-0.06	1.27	-0.11	0.42	1.53	1.34	0.94	-0.68
eIF2A	0.00	-1.42	-0.70	-1.39	0.64	-2.38	-1.82	-1.47
eIF3A	-0.01	1.27	-0.13	0.66	0.62	0.43	0.14	-1.00
eIF3D	-0.02	-0.55	-0.99	-0.52	0.54	0.39	-0.28	-0.82
eIF3H	-0.04	0.73	-0.34	0.23	0.81	0.51	0.60	-0.46
eIF4A1	-0.02	-1.15	0.05	1.44	0.25	0.88	1.78	-0.86
eIF4B	-0.08	0.21	-0.96	-0.91	-0.56	-2.10	-1.72	-1.87
eIF4E	-0.01	0.17	-0.28	0.90	3.04	1.44	2.23	0.42
eIF4EBP1	-0.05	-0.76	-1.74	-0.43	-0.49	-1.36	0.28	-1.09
peIF4EBP1	-0.04	-1.91	-0.70	0.63	-3.87	-0.43	0.65	-0.54
peIF4EBP1/eIF4EBP1	-0.04	-2.76	0.94	1.04	-3.38	0.93	0.36	0.55
eIF4G	-0.01	0.51	-0.06	0.36	1.10	0.06	0.57	-0.46
eIF4H	0.00	-0.96	-0.01	-0.51	0.27	-0.70	-0.59	-0.31
eIF6	-0.06	-1.56	2.06	0.58	3.25	2.48	1.70	0.49

Table S5 B. Statistical analysis of eIF expression in breast cancer cell lines. p-values as determined by ANOVA applying post-hoc analysis (LSD or Tamhane-T2) as indicated depending on equal variance. Significant values are color coded as shown at the end of the table.

eIF2α (T2)	MCF-7	Tam	MDA-MB-468	HS578T	SkBr3	MDA-MB-231	UACC3199	T47D
MCF-7		0.984	0.223	1.000	0.286	0.062	0.105	1.000
Tam	0.623		1.000	0.998	0.618	0.481	0.340	0.942
MDA-MB-468	1.000	0.977		0.993	0.084	0.094	0.064	0.990
HS578T	1.000	1.000	1.000		0.136	0.037	0.054	0.999
SkBr3	0.586	0.941	0.438	0.978		0.866	0.991	1.000
MDA-MB-231	0.036	0.197	0.299	0.943	1.000		1.000	1.000
UACC3199	0.723	0.985	0.552	0.991	1.000	1.000		1.000
T47D	1.000	0.832	1.000	1.000	0.355	0.059	0.499	peIF2α (T2)

peIF2α/eIF2α (T2)	MCF-7	Tam	MDA-MB-468	HS578T	SkBr3	MDA-MB-231	UACC3199	T47D
MCF-7		0.929	1.000	1.000	0.977	0.969	1.000	1.000
Tam	< 0.001		0.991	1.000	1.000	1.000	1.000	0.890
MDA-MB-468	0.002	0.006		1.000	0.992	0.991	1.000	1.000
HS578T	< 0.001	0.872	0.005		1.000	1.000	1.000	1.000
SkBr3	0.004	< 0.001	< 0.001	< 0.001		1.000	1.000	0.893
MDA-MB-231	< 0.001	0.001	< 0.001	< 0.001	< 0.001		1.000	0.892
UACC3199	< 0.001	0.112	< 0.001	0.062	< 0.001	0.019		0.989
T47D	< 0.001	0.864	0.002	0.720	< 0.001	< 0.001	0.123	eIF2A (LSD)

eIF3A (T2)	MCF-7	Tam	MDA-MB-468	HS578T	SkBr3	MDA-MB-231	UACC3199	T47D
MCF-7		0.990	1.000	0.781	0.001	0.891	1.000	0.826
Tam	0.871		0.969	1.000	1.000	1.000	0.996	0.619
MDA-MB-468	1.000	1.000		0.793	0.791	0.970	1.000	0.927
HS578T	1.000	1.000	1.000		1.000	1.000	0.950	0.225
SkBr3	1.000	0.910	0.986	0.994		1.000	0.590	0.390
MDA-MB-231	0.955	0.391	0.996	0.995	1.000		0.999	0.383
UACC3199	1.000	1.000	1.000	1.000	0.999	0.998		0.657
T47D	1.000	1.000	1.000	1.000	0.973	0.983	1.000	eIF3D (T2)

eIF3H (LSD)	MCF-7	Tam	MDA-MB-468	HS578T	SkBr3	MDA-MB-231	UACC3199	T47D
MCF-7		0.002	0.151	0.209	< 0.001	0.012	0.005	0.047
Tam	0.578		< 0.001	0.046	0.751	0.373	0.581	< 0.001
MDA-MB-468	1.000	0.626		0.018	< 0.001	0.001	< 0.001	0.584
HS578T	0.046	0.028	0.103		0.015	0.210	0.109	0.005
SkBr3	0.992	0.344	0.999	0.097		0.197	0.352	< 0.001
MDA-MB-231	0.003	0.271	0.002	0.825	0.244		0.709	< 0.001
UACC3199	< 0.001	0.125	< 0.001	0.997	0.007	0.002		< 0.001
T47D	0.009	1.000	0.010	0.010	0.019	< 0.001	< 0.001	eIF4A1 (T2)

eIF4B (T2)	MCF-7	Tam	MDA-MB468	HS578T	SkBr3	MDA-MB231	UACC3199	T47D
MCF-7		1.000	0.245	0.757	1.000	0.003	0.022	0.016
Tam	0.918		0.718	0.686	0.986	0.254	0.168	0.177
MDA-MB468	0.998	0.886		1.000	1.000	0.007	0.531	0.394
HS578T	0.001	0.036	0.086		1.000	0.353	0.796	0.598
SkBr3	< 0.001	0.006	< 0.001	0.002		0.338	0.568	0.445
MDA-MB231	0.064	0.181	0.016	0.831	0.022		0.995	1.000
UACC3199	0.016	0.054	0.002	0.123	0.406	0.580		1.000
T47D	1.000	1.000	1.000	1.000	0.446	0.998	0.790	eIF4E (T2)

eIF4EBP1 (T2)	MCF-7	Tam	MDA-MB-468	HS578T	SkBr3	MDA-MB-231	UACC3199	T47D
MCF-7		0.778	0.991	1.000	0.989	0.187	1.000	0.416
Tam	0.224		1.000	1.000	0.999	0.809	0.459	0.993
MDA-MB-468	0.999	0.881		0.998	1.000	1.000	0.987	1.000
HS578T	0.741	0.334	0.985		1.000	0.979	0.995	0.999
SkBr3	0.994	1.000	0.997	0.990		0.803	0.939	0.459
MDA-MB-231	0.999	0.534	1.000	0.941	0.997		0.358	1.000
UACC3199	0.998	0.323	0.925	1.000	0.979	0.979		0.835
T47D	0.945	0.659	1.000	0.789	0.998	1.000	0.983	peIF4EBP1 (T2)

p4EBP1 ratio (T2)	MCF-7	Tam	MDA-MB-468	HS578T	SkBr3	MDA-MB-231	UACC3199	T47D
MCF-7		0.068	0.959	0.321	0.997	0.501	1.000	0.842
Tam	1.000		0.120	0.168	1.000	0.064	0.210	0.131
MDA-MB-468	1.000	1.000		1.000	0.984	1.000	1.000	1.000
HS578T	1.000	1.000	0.587		0.991	1.000	1.000	0.979
SkBr3	0.057	1.000	0.556	0.309		0.990	0.992	0.994
MDA-MB-231	1.000	1.000	0.563	0.321	0.261		1.000	0.999
UACC3199	0.598	1.000	0.549	0.295	0.228	0.244		1.000
T47D	0.990	0.990	0.570	0.333	0.275	0.289	0.260	eIF4G (T2)

eIF4H (LSD)	MCF-7	Tam	MDA-MB-468	HS578T	SkBr3	MDA-MB-231	UACC3199	T47D
MCF-7		< 0.001	0.981	0.026	0.219	0.003	0.011	0.164
Tam	0.008		0.001	0.088	< 0.001	0.316	0.156	0.017
MDA-MB-468	0.005	0.002		0.043	0.252	0.007	0.020	0.210
HS578T	0.989	0.108	0.284		0.003	0.423	0.736	0.404
SkBr3	0.002	0.002	0.374	0.024		< 0.001	0.001	0.021
MDA-MB-231	0.290	0.092	1.000	0.550	1.000		0.639	0.109
UACC3199	0.002	< 0.001	0.999	0.612	0.144	1.000		0.246
T47D	1.000	0,955	0,966	1.000	0,755	0,969	1.000	eIF6 (T2)

p<0.1	p<0.05	p<0.01
